# Competitive and/or cooperative interactions of graphene-family materials and benzo[a]pyrene with pulmonary surfactant: a computational and experimental study

**DOI:** 10.1186/s12989-021-00436-9

**Published:** 2021-12-16

**Authors:** Tongtao Yue, Rujie Lv, Dongfang Xu, Yan Xu, Lu Liu, Yanhui Dai, Jian Zhao, Baoshan Xing

**Affiliations:** 1 Institute of Coastal Environmental Pollution Control, Ministry of Education Key Laboratory of Marine Environment and Ecology, Frontiers Science Center for Deep Ocean Multispheres and Earth System, Ocean University of China, Qingdao, 266100 China; 2 Laboratory for Marine Ecology and Environmental Science, Qingdao National Laboratory for Marine Science and Technology, Qingdao, 266237 China; 3 College of Chemical Engineering, China University of Petroleum (East China), Qingdao, 266580 China; 4 College of Electronic Engineering and Automation, Shandong University of Science and Technology, Qingdao, 266590 China; 5 Stockbridge School of Agriculture, University of Massachusetts, Amherst, MA 01003 USA

**Keywords:** Joint effect, Graphene-family materials, Benzo[a]pyrene, Pulmonary surfactant, Molecular dynamics

## Abstract

**Background:**

Airborne nanoparticles can be inhaled and deposit in human alveoli, where pulmonary surfactant (PS) molecules lining at the alveolar air–water interface act as the first barrier against inhaled nanoparticles entering the body. Although considerable efforts have been devoted to elucidate the mechanisms underlying nanoparticle-PS interactions, our understanding on this important issue is limited due to the high complexity of the atmosphere, in which nanoparticles are believed to experience transformations that remarkably change the nanoparticles’ surface properties and states. By contrast with bare nanoparticles that have been extensively studied, relatively little is known about the interactions between PS and inhaled nanoparticles which already adsorb contaminants. In this combined experimental and computational effort, we investigate the joint interactions between PS and graphene-family materials (GFMs) with coexisting benzo[a]pyrene (BaP).

**Results:**

Depending on the BaP concentration, molecular agglomeration, and graphene oxidation, different nanocomposite structures are formed via BaPs adsorption on GFMs. Upon deposition of GFMs carrying BaPs at the pulmonary surfactant (PS) layer, competition and cooperation of interactions between different components determines the interfacial processes including BaP solubilization, GFM translocation and PS perturbation. Importantly, BaPs adsorbed on GFMs are solubilized to increase BaP’s bioavailability. By contrast with graphene adhering on the PS layer to release part of adsorbed BaPs, more BaPs are released from graphene oxide, which induces a hydrophilic pore in the PS layer and shows adverse effect on the PS biophysical function. Translocation of graphene across the PS layer is facilitated by BaP adsorption through segregating it from contact with PS, while translocation of graphene oxide is suppressed by BaP adsorption due to the increase of surface hydrophobicity. Graphene extracts PS molecules from the layer, and the resultant PS depletion declines with graphene oxidation and BaP adsorption.

**Conclusion:**

GFMs showed high adsorption capacity towards BaPs to form nanocomposites. Upon deposition of GFMs carrying BaPs at the alveolar air–water interface covered by a thin PS layer, the interactions of GFM-PS, GFM-BaP and BaP-PS determined the interfacial processes of BaP solubilization, GFM translocation and PS perturbation.

**Supplementary Information:**

The online version contains supplementary material available at 10.1186/s12989-021-00436-9.

## Background

Development and application of nanotechnology rely on fabrication and use of nanomaterials. However, the large-scale production and applications of various nanoparticles have increased the risk of human exposure, thus raising scientific and public concern about the nanoparticles’ biosafety [1, 2]. Once inside the body, nanoparticles can induce unexpected effects through interactions with biological systems [3]. The nature of nano-bio interactions depends on the original nanoparticle properties, coatings and the biological environment [4, 5].

Among possible portals, the human lung can be easily accessed by airborne nanoparticles due to its specific properties, such as the thin epithelial barrier and large surface area [6]. Once inhaled, nanoparticles could get through the branching airway to deposit in the alveolar region, where they first interact with the pulmonary surfactant (PS) lining at the alveolar air–water interface and acting as the first barrier against nanoparticles entering the body [7]. Previous studies discovered carbon nanoparticles in the bronchoalveolar lavage of asthmatic children [8]. Once crossing this barrier, inhaled nanoparticles can translocate to extrapulmonary organs such as liver, breast, and even brain, as manifested by previous rat experiments [2, 9]. Continuous efforts have been made to elucidate the mechanisms underlying the nanoparticle-PS interactions, but our understanding on this important issue is limited due to the high complexity of the atmosphere, in which nanoparticles are believed to experience chemical and physical transformations that remarkably change the nanoparticles’ surface properties and states [10]. For example, carbon nanoparticles undergo oxidization and reduction to add functional groups that are beneficial for adsorption of a variety of organic and inorganic pollutants [11, 12]. By contrast with bare nanoparticles that have been extensively studied, relatively little is known about the interactions between PS and inhaled nanoparticles which already adsorb contaminants. Polycyclic aromatic hydrocarbons (PAHs) are a typical group of persistent organic pollutants generated from natural and manmade combustion processes, during which some carbon nanoparticles can also be produced and show high capacity of adsorbing PAHs through π–stacking interactions [12]. Also, PAHs are good carbon sources for synthesis of carbon nanoparticles using the bottom-up fabrication method [13]. Therefore, carbon nanoparticles in atmosphere could coexist with PAHs.

Graphene is a typical carbon nanomaterial with fascinating physicochemical properties making it a popular material for wide applications [14]. In spite of its excellent performance and wide potential applications, the inhalation toxicity of graphene-family materials (GFMs) has been extensively debated. For example, biophysical function of PS can be perturbed by inhaled GFMs through rigidifying the PS layer [15], increasing the PS layer compressibility [16], and interfering with the normal PS arrangement [17]. However, majority of previous studies focused on single analyte exposure [16, 18, 19], thereby overlooking the complexity of atmosphere chemical composition. For GFM-PAH mixtures inhaled and deposited in alveoli, a typical nano-bio interface is formed including the graphene, adsorbed PAHs, and PS elements composed of lipids and proteins, distinct from that of bare GFMs as previously studied [15–17]. Considering the high capacity of GFMs to adsorb PAHs, we hypothesized that a large number of PAHs could be transported by inhaled GFMs into alveoli, where their joint interactions with PS could induce the release of PAHs to increase their bioavailability [20, 21]. On the other hand, GFMs adsorbed with PAHs may interfere with the ultrastructure and biophysical function of PS through competitive adsorption and exchange of PAHs and PS at the nano-bio interface. Translocation of GFMs across the PS layer may also be altered by PAH adsorption through change of the GFM-PS interactions.

Computational modeling has begun to complement experimental results for deeper understanding of nano-bio interactions [22–24]. Here, we combined molecular dynamics (MD) simulations and experiments to investigate the joint interactions between PS and GFMs with coexisting benzo[a]pyrene (BaP). The objectives were to (1) examine the gaseous adsorption of BaP on GFMs as affected by the BaP concentration, molecular agglomeration, and graphene oxidation; (2) probe the solubilization of BaP by PS that induces the increase of the BaP bioavailability; and (3) reveal the mechanisms underlying competitive interactions among GFMs, BaP and PS that modulate the processes including BaP solubilization, GFM translocation, and PS perturbation. These mechanistic investigations will advance our molecular level understanding on the joint effects of airborne carbon nanoparticles carrying contaminants at membrane-based biological interfaces.

## Results

### BaP agglomeration and adsorption on GFMs

**Fig. 1:**
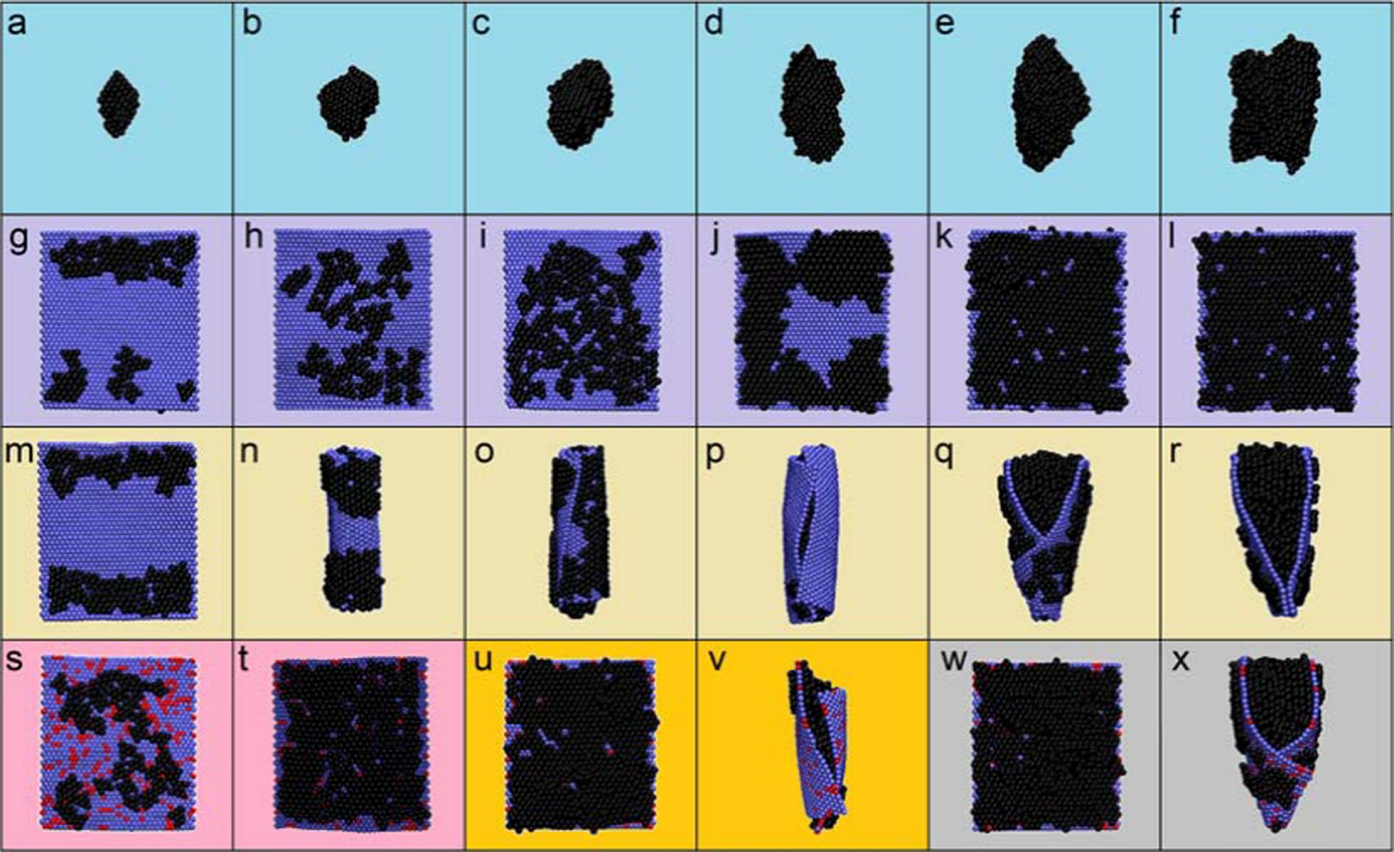
Systematic scan of the BaP agglomeration and adsorption on GFMs. **a**–**f** Simulated aggregates of BaPs of different numbers. **g**–**l** Adsorption of dispersed BaPs on graphene. **m**–**r** Adsorption of aggregated BaPs on graphene. The number of BaPs is respectively 40 (**a**, **g**, **m**), 80 (**b**, **h**, **n**), 120 (**c**, **i**, **o**), 160 (**d**, **j**, **p**), 200 (**e**, **k**, **q**) and 240 (**f**, **l**, **r**). **s**–**x** Adsorption of 80 (**s**, **t**), 200 (**u**, **v**) and 240 (**w**, **x**) BaPs respectively in dispersed (**s**, **u**, **w**) and agglomerated (**t**, **v**, **x**) states on graphene oxide. In each snapshot, BaPs are displayed in black, graphene nanosheet is displayed in blue, and red points represent graphene oxidation

To capture the details at the molecular level while extending time and length scales of MD simulations to be appropriate for studies of BaP agglomeration, adsorption on GFMs, and their joint interactions with PS, the coarse-grained (CG) martini force field was adopted [25]. CG models of component molecules used in MD simulations are shown in Additional file 1: Fig. S1 (detailed description of models and simulation method was given in Methods). Before evaluating the joint effects of PS interactions with GFMs and adsorbed BaPs, we firstly examined the equilibrium states of BaPs in simulated atmospheric environment. It has been demonstrated that PAH molecules are present in atmosphere as vapor and particulate phases [26, 27], and they could adsorb onto carbon nanoparticles [28]. For BaPs initially dispersed in a gaseous box, they were found to form agglomerates of irregular shapes (Fig. 1). Continuous aggregation and disaggregation being accompanied with inner structural rearrangement occurred as reflected in fluctuations of the BaP-BaP interaction energy before reaching equilibrium (Additional file 1: Fig. S2). Relative humidity was considered by adding water molecules into the simulation box. By comparing time sequences of typical snapshots and evolutions of the BaP-BaP interaction energy, humidity barely affected the equilibrium structure but promoted and stabilized BaP agglomeration (Additional file 1: Fig. S3), possibly due to liquid bridging forces which enhanced the agglomeration velocity as previously reported [29].

Next, dispersed BaPs were mixed with a graphene nanosheet of 10.8 nm × 14.4 nm in lateral size, and all BaPs adsorbed on graphene (Fig. 1). Depending on the BaP concentration, single- and multi-layered adsorption structures were formed. The whole process can be described as two stages, as revealed in time evolutions of the graphene-BaP interaction energy (Additional file 1: Fig. S4). In the first stage featured with a sudden decrease of the graphene-BaP interaction energy, dispersed BaPs rapidly diffused and got contact with graphene. Subsequently, the adsorbed BaPs rearranged their positions and orientations to acquire more favorable contacts with both the graphene and neighboring BaPs, as reflected in slower decreases of the interaction energy (Additional file 1: Fig. S4).

For BaPs in the particulate phase encountering graphene, interestingly, they were found to stimulate graphene scrolling into tubular nanostructures (Fig. 1). That was similar to previous findings of the graphene scrolling guided by carbon nanotubes [30], iron nanowires [31], fullerene clusters [32], even nanodroplets of ionic liquid [33]. The graphene scrolling on BaPs was driven by their dispersive interactions, but cost a finite graphene bending energy. Thus, larger BaP agglomerates were easier to scroll graphene by acquiring more favorable contacts and costing lower energy of graphene deformation (Additional file 1: Fig. S5). Similar nanostructures were formed for BaP adsorption on graphene oxide (Fig. 1), and humidity barely affected the equilibrium structures of BaP adsorption on GFMs (Additional file 1: Fig. S6).

### Solubilization of BaPs by PS

**Fig. 2:**
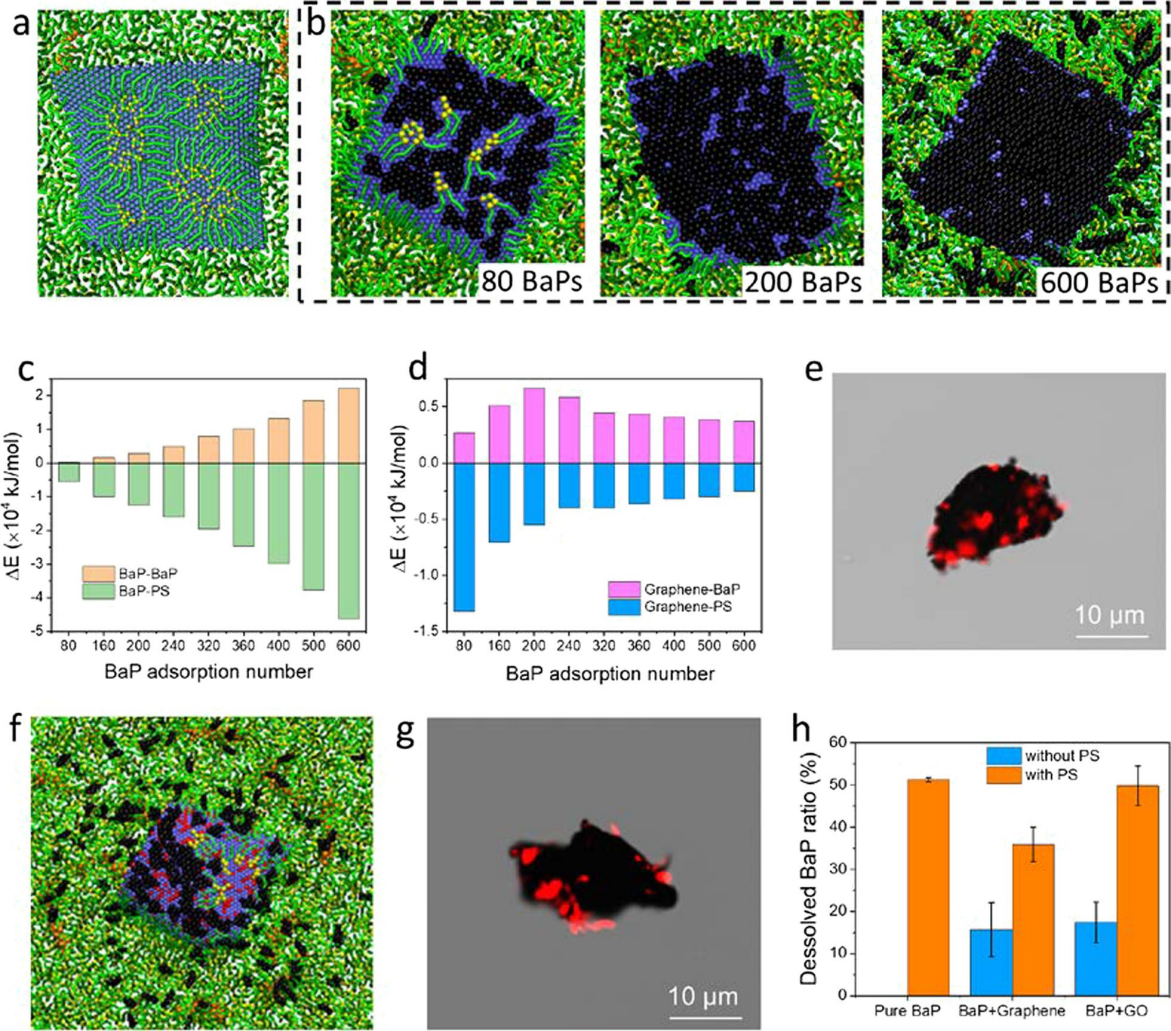
Solubilization of BaPs by PS examined by MD simulations and laboratory experiments. **a** Simulated snapshot of PS adsorption on a bare graphene nanosheet. Lipid tails are diaplayed in green with headgroups in yellow. Water molecules are not displayed for clarity. **b** Snapshots of PS interactions with graphene and adsorbed BaPs. **c** Changes of the BaP-BaP and BaP-PS interaction energies as a function of the total number of BaPs adsorbed on graphene. **d** Changes of the graphene-BaP and graphene-PS interaction energies as functions of the adsorbed BaP number. **e** Confocal imaging of PS adsorption on graphene. Black color represents graphene of approximately 20 μm in lateral size. Red color represents adsorbed PS molecules. **f** Simulated snapshot showing that more BaPs adsorbed on graphene oxide were solubilized by PS. **g** Confocal imaging of PS adsorption (colored in red) on graphene oxide, which is approximately 25 μm in lateral size. **h** Ratio of dissolved BaPs adsorbed on graphene and graphene oxide in the Curosurf solution. In the solubilization experiments, the concentrations of BaP, graphene/graphene oxide and PS were 5, 10, 40 mg/L, respectively, and the sample was incubated at 37 ℃ for 48 h

Given high capacity of GFMs adsorbing BaPs in atmosphere, we speculated that a large number of BaPs are carried by inhaled GFMs into alveoli, where the BaP’s bioavailability can be increased through interactions with PS [20]. Shown in Fig. 2a is a typical snapshot of PS interactions with a bare graphene nanosheet (continuous process is given in Additional file 1: Fig. S7a). As is seen, a number of PS molecules were extracted from the layer and formed inverse micelles on graphene, which can be regarded as coronas altering graphene’s original surface properties and influencing the fate of graphene in subsequent fluids [17, 34]. In addition to PS extraction on the upper graphene surface, molecules underneath the graphene were also perturbed and arranged in higher orders due to attractive interactions with the planar graphene surface (Additional file 1: Fig. S7b, c). MD simulations described the process as the graphene adhesion stage and PS extraction stage, each generating 11.7 kJ/mol and 7.9 kJ/mol decreases of the graphene-PS interaction energy (Additional file 1: Fig. S7d). To differentiate the role of PS components in graphene deposition and PS extraction, the energy of interactions between graphene and different PS molecules was calculated (Additional file 1: Fig. S7e). Combining typical snapshots, it was clear that only lipid components (mostly DPPC and POPG) were extracted by graphene, while nearly no contact between graphene and proteins was observed. When more BaPs adsorbed on graphene, there was an increasing number of BaPs released from graphene and solubilized by PS (Fig. 2b, detailed processes are given in Additional files 2 and 3: Movies S1 and S2), generating more increase in the BaP-BaP interaction energy and decrease in the BaP-PS interaction energy (Fig. 2c and Additional file 1: Fig. S8). We also calculated changes in the graphene-BaP and graphene-PS interaction energies (Fig. 2d), and found that change in the graphene-BaP interaction energy increased only when the number of adsorbed BaPs was less than 240. When more BaPs were adsorbed to reach a multi-layered adsorption state, the energy change contrarily decreased. Less PS molecules were extracted by graphene-BaP complexes than bare graphene, as reflected in the lower decrease of the graphene-PS interaction energy (Fig. 2d). PS adsorption on graphene was experimentally confirmed from confocal imaging (Fig. 2e, details are found in the Methods).

For single-component BaP agglomerates deposited at the PS layer, they were completely solubilized by PS (Additional file 1: Fig. S9a and Additional file 4: Movie S3), as evidenced by a rapid increase in the BaP-BaP interaction energy and a decrease in the BaP-PS interaction energy (Additional file 1: Fig. S9b). The BaP-PS interaction was more energetically favorable than the BaP-BaP interaction, and should be the driving force for BaP solubilization. Among PS components, lipids (DPPC and POPG) played a primary role in BaP solubilization, according to their interaction energy (Additional file 1: Fig. S9c). Compared with pristine graphene, more BaPs were released from graphene oxide and dissolved by PS (Fig. 2f). Upon release of BaPs, PS molecules were extracted and expelled more BaPs into the PS layer. The strong adsorption of PS molecules on graphene oxide was also experimentally observed through confocal imaging (Fig. 2g). Solubilization experiments were conducted to further investigate solubilization of BaPs by PS (Fig. 2h). In the absence of PS, the amount of BaP adsorption on graphene was experimentally measured as 86.9 ± 24.0 mg/g, which was larger than 78.5 ± 31.85 mg/g for BaP adsorption on graphene oxide of lower hydrophobicity. Consistent with the simulation results, BaPs were highly solubilized by 51.2% in the Curosurf solution (details are found in the Methods). By contrast with pristine graphene, the solubilization was higher for BaPs adsorbed on graphene oxide, due to the lower strength of their interactions as revealed by MD simulations.

### PS perturbation by GFM-BaP complexes

**Fig. 3:**
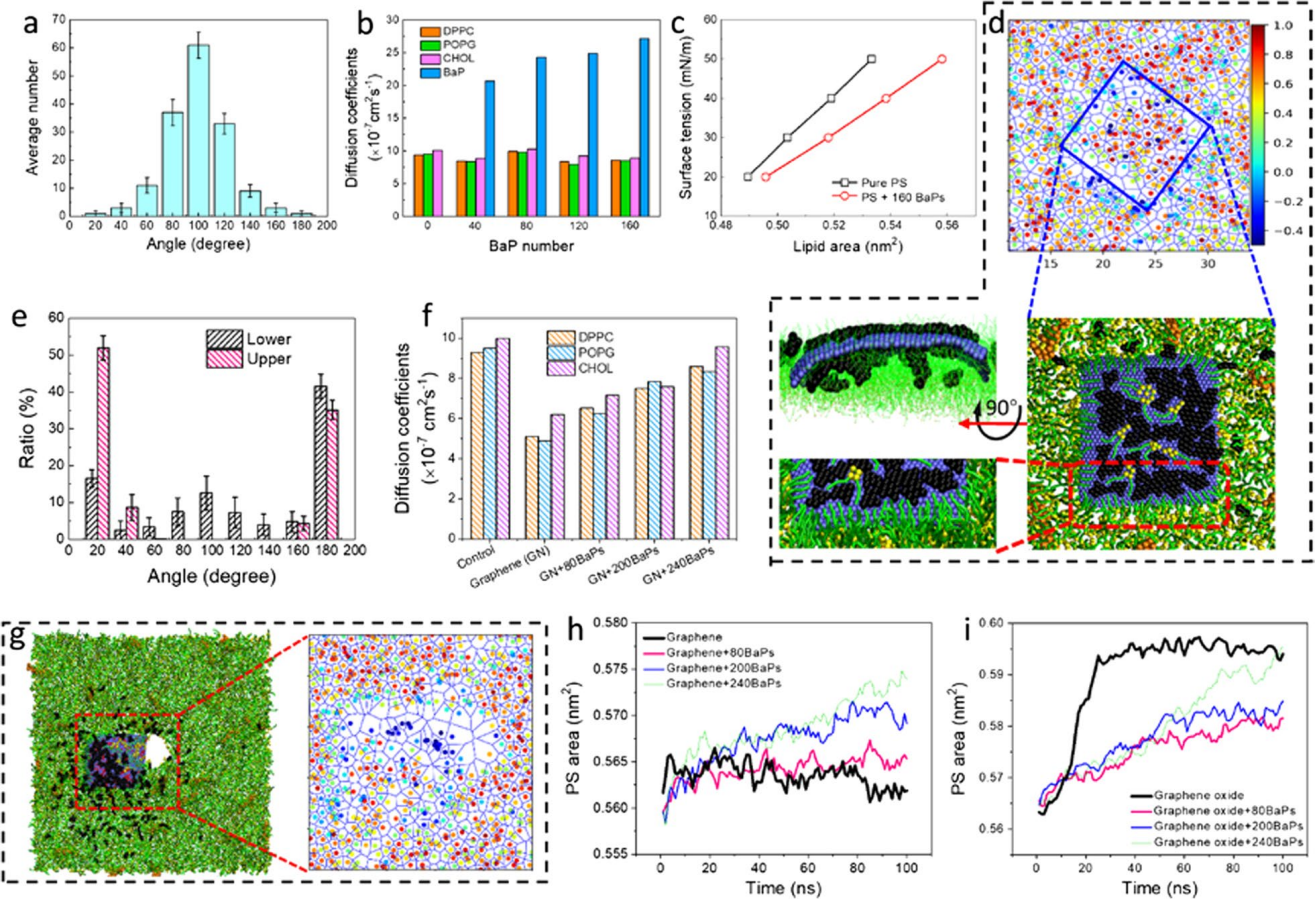
PS perturbation by solubilized BaPs and GFM-BaP complexes. **a** Distribution of the angle between solubilized BaPs and the PS layer. **b** Diffusion coefficients of BaPs and different components of PS. **c** Surface tension of the PS layer as a function of the lipid area as affected by solubilized BaPs. **d** The calculated order parameters for lipids are used to color the Voronoi lattice, with the local structure from different views illustrating perturbed PS arrangement by graphene-BaP complex. **e** Orientation of BaPs above and below the graphene surface. **f** Impact of graphene and graphene-BaP complexes on the diffusion coefficients of PS. **g** Typical snapshot and local order parameter diagram showing enhanced PS perturbation by graphene oxide with 240 adsorbed BaPs. **h**, **i** Time evolutions of the PS area deposited with graphene and graphene oxide adsorbed with BaPs of different numbers

Early deposition of BaP agglomerates induced slight PS perturbation, which disappeared as dispersion of BaPs inside the layer (Additional file 1: Fig. S9a). Solubilized BaPs aligned vertically in the PS layer to acquire more favorable contacts with PS (Fig. 3a). We measured the mean square displacement (Additional file 1: Fig. S10), from which the diffusion coefficients of PS components and BaPs were calculated. Compared to the intact PS layer, deposition of BaPs, which diffused at a higher rate, barely affected the PS liquidity (Fig. 3b). We calculated surface tension of the PS layer (*γ*) as a function of the PS area (*A*), from which the PS layer compressibility, defined as $\kappa { = (}\partial {\text{A/}}\partial \gamma {\text{)/A}}$, was estimated < 0.01 m/mN, similar to that measured by previous experiments [35]. Dissolved BaPs apparently increased the PS layer compressibility (Fig. 3c).

For a bare graphene nanosheet deposited at the PS layer, the PS perturbation was mainly reflected as extraction of PS molecules forming inverse micelles on graphene (Additional file 1: Fig. S7), but a distinct interface was formed between graphene-BaP mixtures and PS. As shown in the order parameter diagram for lipids close to the graphene-BaP complex (Fig. 3d), surrounding PS molecules climbed onto graphene, while those beneath the mixture were aligned in higher orders. We calculated the angles between surfaces of the PS layer and BaPs respectively above and below graphene (Fig. 3e and Additional file 1: Fig. S11). BaPs on the upper graphene surface kept lying flat during the simulation, while those at the graphene-PS interface were partially detached and vertically immersed in the PS layer to enhance the lipid ordering. Graphene adsorbed with more BaPs showed similar PS perturbation (Additional file 1: Fig. S12).

We calculated the diffusion coefficients of PS components as affected by graphene and graphene-BaP complexes. Apparently, deposition of a bare graphene nanosheet strongly reduced fluidity of PS (Fig. 3f), while BaPs solubilized in PS barely affected the PS diffusivity (Fig. 3b). For graphene-BaP mixtures deposited at the PS layer, the restraining effect of graphene on the PS diffusivity was alleviated by adsorbed BaPs. We interpret that it was the strong attraction between graphene and PS to restrain diffusion of surrounding PS molecules. When the original surface of graphene was covered by BaPs, the direct contact between graphene and PS was sterically hindered to alleviate the restraining effect on PS diffusivity.

Curled graphene encapsulating BaPs interacted with the PS layer in a different pathway. They were horizontally immersed in the PS layer to induce PS perturbation (Additional file 1: Fig. S13), similar to that of rod-like nanoparticles and carbon nanotubes as probed in previous studies [36, 37]. The energy of both BaP-PS and graphene-PS interactions decreased, while the energy of BaP-BaP and graphene-BaP interactions slightly increased (Additional file 1: Fig. S13), suggesting that BaPs, especially those encapsulated inside curled graphene, were barely released due to segregation by curled graphene from contact with PS.

Deposition of a graphene oxide nanosheet induced pores in the PS layer (Fig. 3g and Additional file 5: Movie S4), regardless of BaP adsorption (Additional file 1: Fig. S14). For a bare nanosheet of graphene oxide, it rapidly adhered to and pierced through the PS layer from one corner (Additional file 1: Fig. S15). Compared to pristine graphene, graphene oxide of lower hydrophobicity is easier to repel PS molecules and get more favorable contacts with water, thus opening a pore underneath [16]. We calculated the average PS area under different conditions and found different trends of PS layer expansion induced by graphene oxide, graphene, graphene oxide-BaP and graphene-BaP complexes. First, no expansion of the PS layer was induced by graphene (Fig. 3h), while a marked increase of the PS area was induced by graphene oxide (Fig. 3i), suggesting formation of a hydrophilic pore. For graphene adsorbed with BaPs, there was a slight increase of the PS area (Fig. 3h), suggesting release of partial BaPs into the layer to expand it. More BaPs were released from graphene oxide, generating higher increase of the PS area (Fig. 3i). Upon release of BaPs, more PS molecules were extracted, which in turn expelled more BaPs to generate additional decreases in both the graphene oxide-PS and BaP-PS interaction energies (Additional file 1: Fig. S16).

Earlier experiments reported that GFMs can be activated to generate radical contents which caused lipid oxidation and subsequent membrane damage [38, 39]. To elucidate whether PS molecules were oxidized by GFMs in the current systems, malondialdehyde (MDA) as typical peroxidation product of phospholipids was detected through measuring absorbance at wavelength of 532 nm using a commercial test kit (MDA assay kit (TBA method), Nanjing Jiancheng, China). Nearly no malondialdehyde was generated during PS interactions with GFMs (Additional file 1: Fig. S17), suggesting that the PS oxidation induced by GFMs could be neglected.

### GFM translocation across the PS layer

**Fig. 4:**
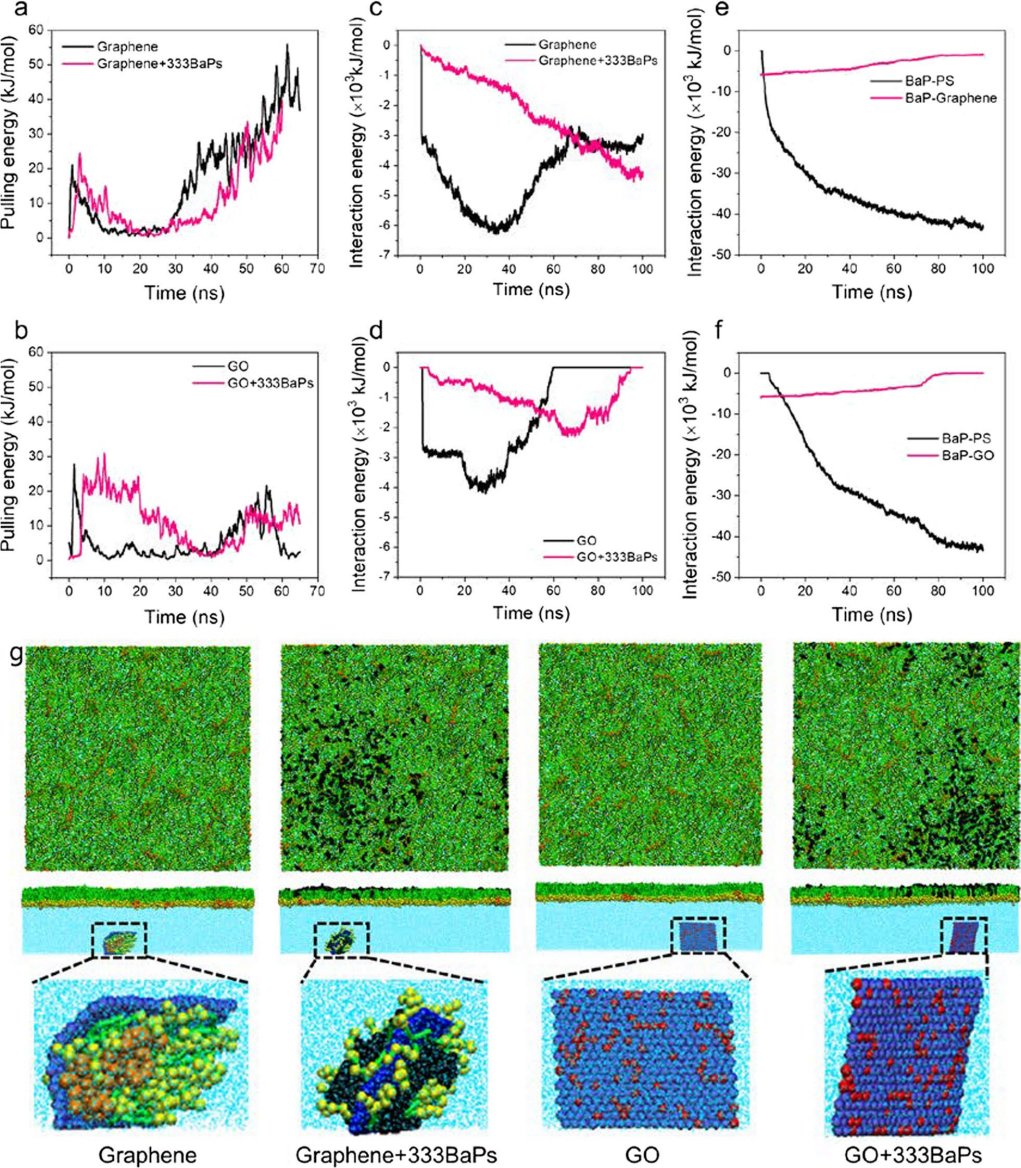
GFM translocation across the PS layer as affected by BaP adsorption. **a**, **b** Time evolutions of the pulling energy for translocation of graphene and graphene oxide with and without adsorption of 333 BaPs. **c**, **d** Time evolutions of the graphene-PS and GFM-PS interaction energies. **e**, **f** Time evolutions of the BaP-PS and BaP-GFM interaction energies. **g** Snapshots showing the final translocation states of graphene and graphene oxide as affected by BaP adsorption

GFMs, owing to the two dimensional plate-like shape, were found to enter cells through directly piercing through the cell membrane [40, 41]. Different from the cell membrane of a lipid bilayer incorporated with various proteins, the PS film is a monolayer of lipids and four types of proteins lining at the air–water interface. We expected different modes of translocation of inhaled GFMs through the PS layer, and the process might be influenced by graphene oxidation and BaP adsorption. To simulate inhalation, an external force was exerted on GFM to pull it along the PS layer normal direction at a constant velocity 0.1 nm/ns, close to the human inhalation velocity as estimated before [15]. Translocation of graphene oxide was more energetically favorable than graphene (Fig. 4a, b), because PS molecules can adsorb on the hydrophobic graphene to impede its translocation, while graphene oxide of lower hydrophobic acquired more favorable contacts with water through translocation. Adsorbed BaPs facilitated translocation of graphene (Fig. 4a), but the translocation of graphene oxide was contrarily retarded (Fig. 4b). That was because BaPs adsorbed on graphene segregated its favorable contact with PS (Fig. 4c, g), while the graphene oxide’s surface hydrophobicity was increased by adsorbed BaPs to enhance attractive interactions with PS (Fig. 4d, g). After translocation, all BaPs adsorbed on graphene oxide were released into the PS layer (Fig. 4e, g), while partial BaPs and PS molecules were carried by graphene to enter the subsequent phase (Fig. 4f, g), where they might encounter different types of cells. To examine the interaction of cell membrane with BaP-adsorbed GFMs, a multi-component lipid bilayer composed of saturated DPPC, unsaturated DOPC and cholesterol molecules was employed for MD simulations. It was observed that graphene can readily pierce through the membrane, inside which most adsorbed BaPs were solubilized and partitioned into the disordered membrane domain (Additional file 1: Fig. S18a). By contrast, graphene oxide with lower hydrophobicity did not enter the membrane in the finite simulation time, but the adsorbed BaPs were completely released into the membrane (Additional file 1: Fig. S18b).

### Extraction of PS molecules by suspended GFMs affected by BaP adsorption

**Fig. 5:**
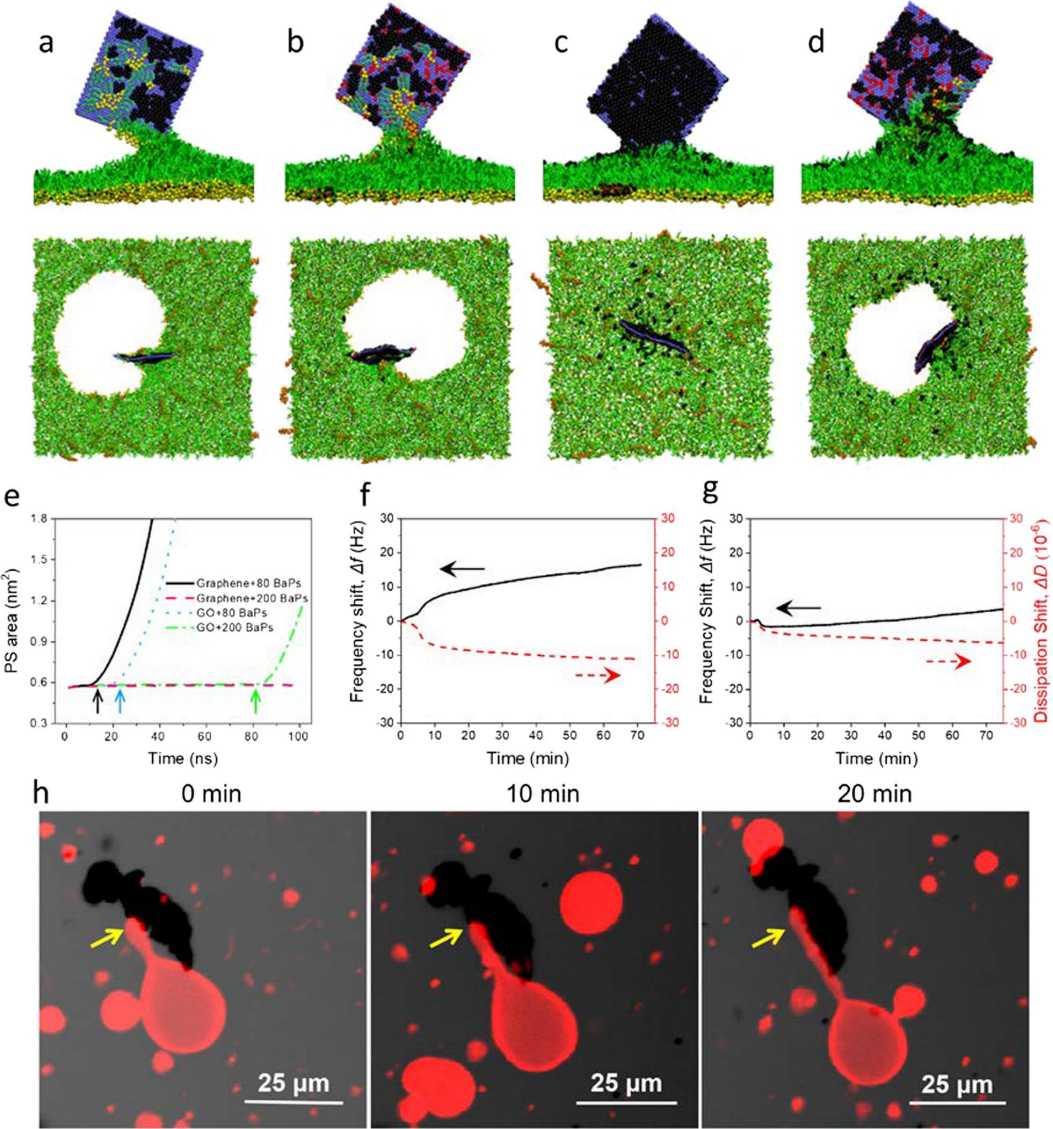
PS extraction and perturbation by GFMs as affected by BaP adsorption. **a** PS extraction by suspended graphene adsorbed with 80 BaPs. **b** PS extraction by graphene oxide adsorbed with 80 BaPs. **c** Graphene adsorbed with 200 BaPs. **d** Graphene oxide adsorbed with 200 BaPs. Each snapshot is displayed from both side and top views for illustration of associated PS extraction, BaP release and PS layer rupture. **e** Time evolutions of the PS area under different conditions. The time points for PS layer rupture are labelled with colored arrows. **f**, **g** LUV interactions with graphene and graphene oxide monitored by QCM-D. The changes of the frequency shift and dissipation shift show the lipid extraction by graphene and graphene oxide. The concentrations of graphene/graphene oxide suspensions were 20 mg/L and the flow rate of QCM-D was fixed at 0.1 mL/min for more than 20 min. **h** Attachment of GUV on graphene and the induced vesicle perturbation as a function of incubation time (0–20 min) as imaged by LCSM. Graphene suspension was diluted to 50 mg/L by 0.1 M glucose, and observed for at least 20 min

**Fig. 6:**
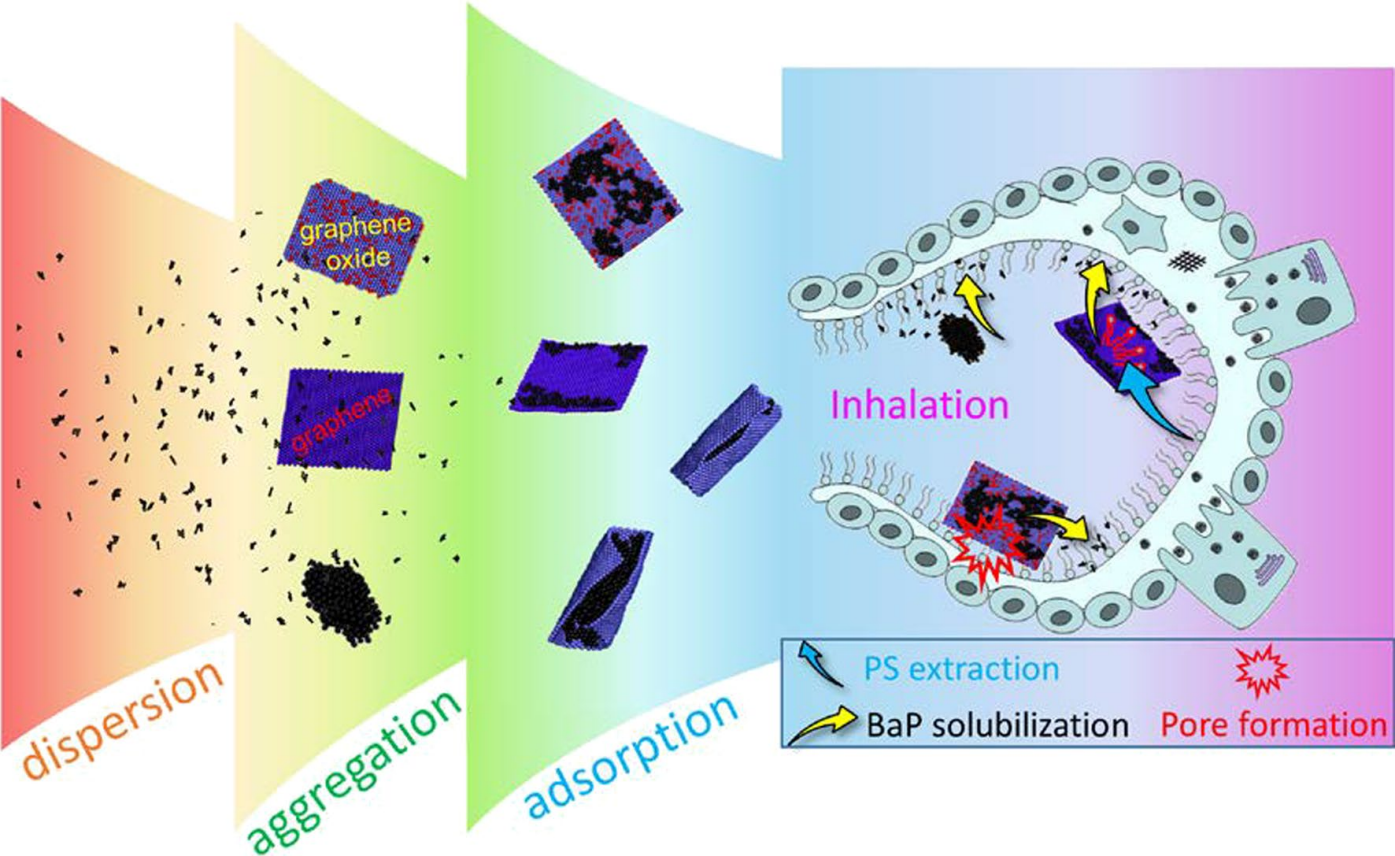
Schematic illustration of the gaseous agglomeration and adsorption of BaPs on GFMs, and their deposition at the alveolar region, where the competition of interactions among GFMs, BaPs and PS determines the interfacial processes including BaP solubilization, GFM translocation and PS perturbation

Under certain conditions of GFM suspension or slow PS layer translocation, PS molecules can be spontaneously extracted from the layer and form inverse micelles on GFM surfaces [17, 37]. For the suspended graphene nanosheet adsorbed with 80 BaPs, PS molecules were rapidly extracted and repelled adsorbed BaPs to form more compact stacking on graphene (Fig. 5a). In consequence of the destructive PS extraction, the PS layer ruptured at *t* = 10 ns (Fig. 5e), and the pore edge was composed of DPPC and POPG molecules with their hydrophilic headgroups pointing to surrounding water (Additional file 1: Fig. S19). By contrast, less PS molecules were extracted by graphene oxide (Fig. 5b and Additional file 1: Fig. S20), and the PS layer ruptured 10 ns later (Fig. 5e). When 200 BaPs adsorbed on graphene to reach a saturated adsorption state, no PS was extracted in the finite simulation time, despite release of few BaPs into the layer (Fig. 5c and Additional file 1: Fig. S20). By contrast for graphene oxide, more BaPs were released, thus offering space to extract PS molecules (Fig. 6d, Additional file 1: Fig. S20 and Additional file 6: Movie S5), and the PS layer ruptured at *t* = 82 ns (Fig. 5e). By contrast with bare GFMs, pre-adsorption of BaPs decreased the available space for PS extraction, and the induced PS depletion was alleviated.

In cycles of respiration, the normal PS layer surface tension varies from tens mN/m to near zero [35], and the interfacial PS layer is connected with other PS membranes in the subphase to help maintain the PS layer tension at lower values. Previous studies have demonstrated higher extent of PS extraction under lower surface tension [17]. In our simulations, the relatively high surface tension of 60 mN/m represented an over-expanded state, and GFMs showed high capacity of extracting PS molecules to further increase the layer surface tension. If the layer relaxation time is slower than the rate of PS depletion, the PS layer would rupture. Such effect of PS depletion induced by GFMs could be amplified by increasing the GFM size and concentration.

The destructive extraction of PS molecules was experimentally verified and quantitively measured using QCM-D (details are found in Methods). First, the cover of large unilammelar vesicles (LUVs) on the Au crystal sensor was confirmed by the changes of frequency shift *∆f* and dissipation shift *∆D* (Additional file 1: Fig. S18). When GFM suspension flowed through the LUV layer, *∆f* showed an upward trend due the mass loss (Fig. 5f). By contrast, *∆f* increased less when graphene oxide suspension of the same concentration flowed through the layer (Fig. 5g), suggesting lower capacity of extracting PS molecules for graphene oxide of lower hydrophobicity. After detecting that graphene of higher hydrophobicity interacted with lipids more strongly than graphene oxide, we employed fluorescently labeled giant unilammelar vesicles (GUVs) to directly observe their interactions using LCSM (Fig. 5h, the details are found in Methods). As is seen, GUVs stably bound to graphene, and a tubular structure protruded from the area of vesicle attachment on graphene, strongly suggesting membrane perturbation induced by graphene.

## Discussion

Understanding how inhaled nanoparticles deposit in alveoli and firstly interact with PS lining at the alveolar air–water interface is of importance to elucidate the toxic effect and subsequent fate of nanoparticles inside the body [42]. Previous experimental and simulation studies have revealed twofold outcomes of the nanoparticle-PS interactions. On one hand, the ultrastructure and physiological functions of the endogenous PS can be perturbed by nanoparticles to induce the toxicological effect [36, 43, 44]. On the other hand, the subsequent fate of nanoparticles inside the body is influenced by forming PS coronas to mask the original nanoparticle surface properties [34]. However, most previous studies considered single analyte exposure [16, 18, 19], thereby overlooking complexity of the atmosphere in which nanoparticles are believed to experience transformations with their physicochemical properties and states distinct from their nascent counterparts. It is believed that the inhalation toxicity of nanoparticles is not only from their own harmful nature but also from their adsorbed toxic substances [45, 46]. We consider one typical scenario of incomplete combustion of organic materials, during which both PAHs and carbon nanoparticles can be generated with high concentrations [47]. The PAH molecules in atmosphere are evidenced to be present as vapor and particulate phases [26, 27], and they could adsorb onto carbon nanoparticles [28]. Other types of contaminants, such as polychlorinated biphenyls and heavy metals, could also be adsorbed by airborne nanoparticles [48]. This study aims to elucidate the joint effects of PS interactions with GFMs and coexisting BaPs. Processes including the gaseous agglomeration and adsorption of BaPs on GFMs, and their joint interactions with PS under different conditions have been considered as illustrated in Fig. 6.

Firstly, BaPs in atmosphere spontaneously agglomerate and adsorb on GFMs to form single-layered, multi-layered and curled nanocomposite structures. That is different from the aqueous adsorption process, during which only the single-layered adsorption can be formed [12]. Relative humidity barely affects the equilibrium structures of BaP agglomeration and adsorption on GFMs, but promotes BaP agglomeration, possibly due to liquid bridging forces as previously reported [29]. Upon inhalation and deposition of GFMs carrying BaPs in alveoli, the interfacial phenomenon is a result of multiple interactions, including GFM-BaP, BaP-BaP, BaP-PS, GFM-PS, and PS-PS. These interactions generate joint competitive and cooperative effects that can change the arrangement and exchange of different molecules at the nano-bio interface [22, 49]. In particular, our results showed that BaPs are immediately solubilized by PS, because the BaP-PS interaction was more energetically favorable than the BaP-BaP interaction and should be the driving force for BaP solubilization. By contrast with BaPs in direct contact with GFMs, BaPs at the outer adsorption layer are easier to be solubilized by PS, suggesting that more BaPs can be transported by inhaled GFMs into alveoli, where the BaP’s bioavailability is increased through interactions with PS. Besides the energetic competition determining whether and how many BaPs adsorbed on GSMs can be solubilized by PS, the rate of BaP solubilization was higher than that of GFM reaching equilibrium of interactions with PS (Additional file 1: Figs. S7 and S9). Thus, BaPs carried by GFMs can be rapidly solubilized after their deposition at the PS layer. Dissolved BaPs diffused rapidly and aligned vertically in the PS layer to increase the PS compressibility, as measured from the compression isotherm. Note that one of the major biophysical functions of natural PS is to reduce the surface tension of the alveolar air–water interface to prevent alveolar collapse. During exhalation with the alveolar region being compressed, a healthy PS film should have a low compressibility to allow the surface tension to decrease to near-zero with less than 20% area compression [50]. Upon increase of the PS layer compressibility by deposition of BaPs, more area compression would be needed to achieve decrease of the surface tension. It means the PS layer could readily undergo collapse at end of exhalation, leading to respiratory failure.

No apparent phase separation of the multi-component PS layer was observed in the finite simulation time, possibly because the monounsaturated POPG rather than the diunsaturated DOPC or DOPG was adopted in simulations under a relatively high temperature (310 K) [51]. However, natural PS containing both saturated and unsaturated lipid species is known to experience phase segregation, which may influence partitioning of GFMs and adsorbed BaPs in the PS layer. Besides, the PS film undergoes reversable shape transformation during cycled respiration [52], and the solubilized BaPs can be transferred into subsequent fluids to cause other adverse impacts [52, 53]. Considering that BaPs are highly toxic through forming bulky adducts with DNA or producing free radicals that lead to mutation and cancer [53], the interaction of inhaled BaPs with PS is the onset of increasing their bioavailability, following which the toxicity can be exponentially magnified.

It is noted that graphene and graphene oxide behave differently at the PS layer. On one hand, competition between BaP-PS, BaP-GFM and GFM-BaP interactions can be tuned by graphene oxidation, and BaPs adsorbed on graphene oxide of lower hydrophobicity are easier to be solubilized by PS. On the other hand, graphene oxide can easily repel PS molecules and induce a hydrophilic pore to get more favorable with underneath water. That was not observed for graphene, which simply adhered on the PS layer to release part of adsorbed BaPs. PS molecules are extracted by GFMs to induce the PS depletion and layer rupture. Extracted PS molecules form inverse micelles, which can be regarded as PS coronas concealing the original GFM’s surface properties and influencing GFM’s subsequent fate inside the body. For instance, the surface hydrophobicity of GFMs is reduced by PS corona, and adsorbed PS components could enhance or suppress recognition of GFMs by subsequent cell receptors [54]. By contrast with PS coronas formed on bare nanoparticles as previously studied [34, 55–57], pre-adsorption of BaPs is found to inhibit PS corona formation due to their competitive adsorption. Although graphene oxide of lower hydrophobicity extracts less PS molecules, adsorbed BaPs are easier to be solubilized for adsorption of PS. Also, translocation of graphene across the PS layer is facilitated by adsorbed BaPs through segregating it from contact with PS. In contrast, the ease of graphene oxide translocation is reduced by BaPs due to increase of the surface hydrophobicity plus surface passivation. Besides BaP solubilization with resultant increase in bioavailability, binding of BaPs influences the PS layer perturbation induced by GFNs. These results are consistent with a recent experimental finding that, apart from dissolution of toxic BaPs from carbonaceous nanoparticles, binding of BaPs alters the bioreactivity of nanoparticles through eliciting more severe membrane perturbation, endocytosis and oxidative stress [58]. Overall, all these processes including BaP solubilization, GFM translocation and PS perturbation are essentially determined by the competitive and/or cooperative interactions among different components at the nano-bio interface.

## Conclusions

This study systematically explored agglomeration and adsorption of BaPs on GFMs, and their joint interactions with PS lining at the alveolar air–water interface. Depending on the BaP concentration, molecular agglomeration, and graphene oxidation, different nanostructures are formed through BaPs adsorption on GFMs. After deposition of GFMs carrying BaPs into alveoli, competitive and/or cooperative interactions among different components determines the interfacial processes including BaP solubilization, GFM translocation and PS perturbation. Importantly, BaPs either in agglomerates or adsorbed on GFMs are solubilized by PS to increase their bioavailability. Compared to graphene, graphene oxide of lower hydrophobicity releases more BaPs into the PS layer and opens hydrophilic pore to induce severe PS layer damage. PS molecules can be rearranged and extracted by GFMs, and the resultant PS depletion declines with graphene oxidation and BaP adsorption. Translocation of GMFs across the PS layer is affected by BaP adsorption and oxidation through modulating GFM’s surface hydrophobicity plus surface passivation. Carbonaceous nanoparticles are ubiquitous in atmosphere and have the similar graphitic nanostructures to adsorb PAHs. These results have implications for evaluating the combined inhalation toxicity of airborne nanoparticles along with their coexisting toxic contaminants.

## Methods

### Materials

Curosurf extracted from porcine lungs was used in our experiments (Chiesi Pharmaceutici, Parma, Italy), which is a reasonable surrogate of human PS. Graphene was purchased from Graphene supermarket, and graphene oxide was synthesized according to our previous work (detailed process of the graphene oxide synthesis is given in Additional file 1: Text S1) [59]. Scanning electron microscope (SEM), transmission electron microscope (TEM) and atomic force microscope (AFM) imaging were used to characterize GFMs (Additional file 1: Fig. S22, details are given in Additional file 1: Texts S2). It was clear that both graphene and graphene oxide used in our experiments had the typical two-dimensional shape with 10–20 μm in lateral size and < 40 nm in thickness. The C:O ratio was calculated to 93%:7% and 88%:12% respectively for graphene and graphene oxide according to the XPS analysis. BaP was purchased from Aladdin Reagent Company (Shanghai, China). The zwitterionic lipids of 1,2-dioleoyl-sn-glycero-3-phosphocholine (DOPC) and negatively charged 1,2-dioleoyl-sn-glycero-3-[phosphor-rac-(1-glycerol)] (DOPG) were obtained from Avanti Polar Lipids (Alabaster, AL). Although the lipid species adopted in our experiments are different from real components of natural PS, which contains saturated DPPC and monounsaturated POPG as the most abundant, the nature of their interactions with GFMs is similar for explaining the GFM induced PS layer damage. Silicone sheets with a thickness of 0.5 mm and density of 1.2 g/cm^3^ were purchased from Altec (UK) and used for solubilization experiments.

### Solubilization experiment

BaP solubilization by PS was investigated with a passive dosing method avoiding centrifugation (Additional file 1: Fig. S23) [20, 60]. Firstly, silicone sheets were cut into strips of 200 ± 2 mg. These strips were washed three times using acetone and methanol, and seven times with ultrapure water, each lasting for eight hours. The cleaned silicone strips were then stored in a sealed amber bottle with ultrapure water. Graphene, graphene oxide and PS (Curosurf) stock suspensions (graphene and graphene oxide were dispersed in water and sonicated for 10 min by probe sonication (FB750, Fisher Scientific, USA)) were prepared with 500 mg/L, 500 mg/L and 1000 mg/L, respectively. BaP was dissolved in acetone to obtain the stock solution of 1000 mg/L. One piece of cleaned silicone sheet was put into a glass vial, in which BaP solution was then added to reach a concentration of 5 mg/L. After that, graphene, graphene oxide and PS stock suspensions were added to reach concentrations of 10 mg/L, 10 mg/L and 40 mg/L, respectively. The mixed samples were incubated in 37 ℃ for 48 h to allow the systems reach equilibrium state. After incubation, silicon sheets were washed by ultrapure water and wiped to remove the residual water. After washing, silicon sheets were immersed in acetone to extract the BaP for 24 h. Extracted BaP was determined by HPLC according to the previously reported methods [61]. Each group of the solubilization experiment was repeated three times to acquire the average value of BaP solubilization by PS.

### Vesicle preparation

To probe favorable interactions between GFMs and lipids for explaining the GFM induced PS layer damage, large unilamellar vesicles (LUVs) and giant unilamellar vesicles (GUVs) composed of DOPC and DOPG lipids were prepared using the extruding method and gentle hydration procedure, respectively [62]. These two unsaturated lipid species, despite being different from real components of natural PS, were adopted for ease of preparing vesicles, and have been widely used for studies of membrane interactions with nanomaterials [63]. DOPC and DOPG stock solutions were obtained by dissolving lipids in a mixed solution of trichloromethane and methanol (2:1, V/V) and the final concentration was 18 mg/mL and 2 mg/mL, respectively. 50 μL DOPC and 50 μL DOPG solutions were sufficiently mixed in glass vials. Then, lipid solution was dried by nitrogen to remove the organic solvent, and the lipid film was formed at the wall of vials. Further 30-min vacuum-drying was used to remove residual organic solvent. After drying, lipid films were hydrated with ultrapure water for 30 min to form lipid suspension. Prepared lipid suspension was extruded through an extruder for more than 30 times to get LUV stock solution with a PC filter of 100 nm pore size. In the end, LUV stock solution was diluted for 5 times by Tris (10 mM Tris and 150 mM NaCl) to obtain the final LUV solution for use in subsequent experiments. To obtain the fluorescently labelled GUVs, RhB-PE (10 μL, 0.1 mg/mL) was added into the mixture solution of DOPC and DOPG. After drying, the lipid film was hydrated with sucrose solutions (4 mL, 0.1 M) at 40 ℃ for 24 h to form GUV solution. Using sucrose solution can help GUVs maintain osmotic pressure balance with regular spherical shapes for ease of observing interactions with graphene. Subsequent confocal imaging manifested stable spherical shapes for GUVs under current conditions.

### Confocal imaging

Fluorescence images between dyed PS and graphene/graphene oxide were obtained by laser confocal scanning microscope (LCSM, Nikon A1^+^, Nikon, Japan) [64]. In brief, PS and graphene/graphene oxide stock suspensions were mixed and diluted to the final concentration of 40 and 50 mg/L, respectively. The samples were incubated (37 ℃, 150 rpm) for 48 h to reach the equilibrium state. After incubation, 100 μL, 100 μM DiI (1,10-dioctadecyl-3,3,30,30-tetramethylindocarbocyanine perchlorate) (Solarbio, China) fluorescent dye was added into 900 μL samples and incubated at 37 ℃ for 10 min. Dyed samples were washed for three time by PBS (pH = 7.2) and observed by LCSM.

For observation of membrane perturbation induced by graphene, GUV solution was added into a specific peri dish for LCSM with glass bottom. Then, graphene was diluted to 50 mg/L by 0.1 M glucose and added into the dish. Finally, the interactions between GUVs and graphene were examined by LCSM through fluorescence imaging. During each imaging experiment, different views were selected and observed for at least 20 min to acquire the dynamic information of GUV perturbation by graphene.

### QCM-D experiments

Lipid extraction by GFMs was monitored by QCM-D equipped with an Au-coated crystal sensor [62, 65]. In brief, ultrapure water and Tris solution (10 mM Tris and 150 mM NaCl) were flowed through the sensor for 10 min, respectively, to obtain a stable baseline for experiments. Then, LUV solution was flowed through the sensor for about 20 min (at equilibrium of the frequency shift, *∆f*) to form a LUV layer on the surface of crystal sensor. Note that the LUVs maintained their closed, water-containing liposomal structures on the sensor surface, as has been previously demonstrated using the same procedure [63]. After that, ultrapure water and Tris solution were flowed for 10 min, respectively to remove the un-coated LUV of crystal sensor. Finally, 20 mg/L graphene or graphene oxide suspensions were flowed to examine the lipid extraction of graphene and graphene oxide until *∆f* reached equilibrium. The flow rate of QCM-D was fixed at 0.1 mL/min during all experiment process. The QCM-D experiments were repeated three times and similar results were observed.

### Models of component molecules used in simulations

Simulations were based on the molecular dynamics (MD) method and performed using the GROMACS 4.6.7 software package [66]. The widely used Martini coarse-graining method were adopted to prepare the model of each component molecule used in simulations [25]. The PS monolayer of 40 nm × 40 nm in lateral size consists of dipalmitoyl phosphatidylcholine (DPPC) and palmitoyloleoyl phosphatidylglycerol (POPG) with a 7:3 molar ratio, doped with 10 wt% cholesterol, 1.6 wt% surfactant protein (SP)-B, and 1.5 wt % SP-C (Additional file 1: Fig. S1a, b) [44]. All-atom models of the two hydrophobic proteins were obtained from the protein data bank (PDB ID: 2DWF for mini-B and 1SPF for SP-C), and transferred to the corresponding coarse-grained (CG) models using the martinize.py script provided by the Martini force field [67]. In particular, mini-B is a 34-residue peptide composed of the N- and C-terminal helical regions of the full-length 79-residue SP-B, the detailed structure of which has not been obtained. It has been demonstrated that mini-B retains a similar activity of the full-length SP-B [68]. Other two types of proteins (SP-A and SP-D) were not included as they are weakly associated with the PS layer with negligible relation to the PS biophysical function. In addition, to probe how GFMs adsorbed with BaPs interact with the subsequent cell membrane, a lipid bilayer of 22 nm × 22 nm in lateral size was adopted consisting of 612 DPPC, 816 DOPC and 612 cholesterol molecules.

The atomic structure of graphene was reduced to a triangular lattice of SC4-type beads, where every three carbon atoms were modeled as one bead [69]. The angular force constant and the equilibrium angle are $\kappa_{angle}$ = 700 kcal mol^−1^ rad^−2^ and *θ* = 60°, respectively. The bond force constant and equilibrium bond length are $\kappa_{bond}$ = 700 kcal mol^−1^ and *l* = 0.3684 nm, respectively. The dihedral angle force constant, multiplicity and equilibrium angle are $\kappa_{\chi }$ = 3.1 kcal mol^−1^, *n* = 2 and *δ* = 180°, respectively. To represent graphene oxidation, 20% of beads were randomly replaced with the hydrophilic Nda type beads [16, 17]. The CG model of BaP was prepared using the same procedure as graphene. The water slab beneath the PS layer is 9 nm in thickness, containing 667 Na^+^ ions to neutralize the system. The atomic structure of graphene was reduced to a triangular lattice of SC4-type beads, where every three carbon atoms were modeled as one bead (Additional file 1: Fig. S1c) [69]. To represent graphene oxidation, 20% of beads were randomly replaced with the hydrophilic Nda type beads (Additional file 1: Fig. S1d) [16, 17]. The CG model of BaP was prepared using the same procedure as graphene (Additional file 1: Fig. S1e). It should be noted that the GFMs employed in simulations were much smaller than that used in experiments, and simulating large GFMs is rather computationally expensive and still a challenge for current simulations. Nevertheless, the critical molecular events at the nano-bio interface can be reproduced by simulations using nanosheets of graphene and graphene oxide, as has been proved by previous studies [40, 70].

### Modeling BaP agglomeration and gaseous adsorption on GFMs

The PAH concentration in atmosphere generally shows a significant seasonal cycle and varied over a wide range temporally and spatially (ng/m^3^ − μg/m^3^) [71]. For locations of incomplete combustion of fossil fuels, both PAH and carbon nanoparticles of relatively high concentrations could be generated [72]. To mimic this scenario and reduce the time of BaP agglomeration and gaseous adsorption on GFMs to be affordable by simulations in the finite box, BaPs of relatively high concentrations were adopted. Both vapor and particulate phases of BaPs were used to simulate their adsorption on GFMs. Firstly, BaPs were positioned randomly in a gaseous box (50 nm × 50 nm × 50 nm) for simulations of the BaP agglomeration. For gaseous adsorption of vapor BaPs on GFMs, one sheet of graphene or graphene oxide (10.8 nm × 14.4 nm) was randomly mixed with dispersed BaPs. To probe adsorption of particulate BaPs on GFMs, the acquired BaP agglomerates were positioned alongside GFMs. In certain cases, a number of water molecules were added in the simulation box to explore the effect of relative humidity on BaP agglomeration and adsorption on GFMs.

### Modeling PS interactions with BaPs and BaP-GFM mixtures

After acquiring equilibrium configurations of BaP agglomeration and adsorption on GFMs, they were respectively positioned 5 nm above the pre-equilibrated PS layer to explore their interactions. Apart from complete deposition of GFMs carrying BaPs at the PS layer, some inhaled GFMs may adopt the suspending state in the alveolar region, and only partial region of suspended GFMs was allowed to get contact with the PS layer [17]. To probe how the suspended GFMs interact with PS as affected by BaP adsorption, graphene and graphene oxide adsorbed with BaPs were positioned above the PS layer with only bottom sites in close contact with PS, while the top sites were fixed to maintain their suspended states [70].

### Simulation details

Considering that inhaled GFMs deposit in alveoli most probably during the inspiration process, the surface tension was kept constant at 60 mN/m to represent an over expanded state of the PS layer, using the semi-isotropic Berendsen barostat with a coupling constant of $\tau_{{\text{P}}}$ = 4 ps. The system compressibility was set to be 5 × 10^–5^ bar^−1^ in the x–y plane and 0 in the z-direction. Temperature was kept at 310 K by Berendsen coupling with a constant of $\tau_{{\text{T}}}$ = 1 ps. Periodic boundary conditions were applied in the x–y plane, and a dummy wall was set at bottom of the simulation box to prevent mixing between the water and gaseous phases. Nevertheless, a number of water molecules can permeate through the PS layer to enter the gaseous phase and generate relative humidity [73]. A cutoff of 1.2 nm was used for van der Waals interactions, and the Lennard–Jones (LJ) potential was smoothly shifted to zero between 0.9 nm and 1.2 nm to reduce the cutoff noise. For electrostatic interactions, the coulombic potential with a cutoff of 1.2 nm was smoothly shifted to zero from 0 to 1.2 nm. The time step of simulations was 10 fs, with the neighbor list updated every 10 steps. Snapshots were rendered by VMD [74].

## Supplementary Information

**Additional file 1: Text S1**. Synthesis of graphene oxide. **Text S2**. SEM, TEM and AFM imaging of GFMs. **Figure S1**. Coarse-grained models of different component molecules and the simulation system setup. **Figure S2**. Simulated molecular agglomeration of BaPs in atmosphere. **Figure S3**. Effect of relative humidity on BaP agglomeration. **Figure S4**. Simulated adsorption of dispersed BaPs on graphene. **Figure S5**. Time sequences of typical snapshots depicting adsorption of agglomerated BaPs on graphene and the induced graphene curling. **Figure S6**. Effect of relative humidity on BaP adsorption on graphene. **Figure S7**. Deposition of a bare graphene nanosheet on the PS layer. **Figure S8**. Time evolutions of the energy of interactions between different components under different adsorption states. **Figure S9**. Solubilization of agglomerated BaPs by PS. **Figure S10**. The calculated mean square displacement (MSD) for different components of PS and the deposited BaPs of different numbers. **Figure S11**. Distinct orientations of BaPs respectively at the upper and lower surfaces of graphene deposited at the PS layer. **Figure S12**. Ultrastructure perturbation of PS induced by graphene adsorbed with 200 BaPs. **Figure S13**. Joint interactions between the PS layer and curled graphene with encapsulated BaPs. **Figure S14**. Final simulated snapshots from both top and bottom views and the local PS order parameter diagrams. **Figure S15**. Deposition of a bare graphene oxide nanosheet on the PS layer. **Figure S16**. Effects of graphene oxidation and BaP adsorption on joint interactions between graphene, BaP and PS. **Figure S17**. Detection of malondialdehyde. **Figure S18**. MD simulations of cell membrane interactions with GFMs adsorbed with BaPs. **Figure S19**. Typical snapshot showing distribution of PS molecules at the pore edge. **Figure S20**. PS extraction and layer damage induced by graphene as affected by graphene oxidation and BaP adsorption. **Figure S21**. The formation of LUV layer on the QCM-D sensor (Au sensor). **Figure S22**. SEM, TEM and AFM imaging of GFMs. **Figure S23**. Schematic illustration of the solubilization experiment procedure.

**Additional file 2: Movie S1**. Continuous process of graphene carrying 80 BaPs deposited on the PS layer.

**Additional file 3: Movie S2**. Continuous process of graphene carrying 260 BaPs deposited on the PS layer.

**Additional file 4: Movie S3**. Complete solubilization of BaPs in agglomerates by PS.

**Additional file 5: Movie S4**. Deposition of a graphene oxide nanosheet with adsorbed BaPs induced pores in the PS layer.

**Additional file 6: Movie S5**. Molecular exchange of BaP and PS on a suspended graphene oxide nanosheet.

## Data Availability

The datasets used and/or analyzed during the current study are available from the corresponding authors on a reasonable request.

## Abbreviations

GFM: Graphene-family material
PS: Pulmonary surfactant
BaP: Benzo[a]pyrene
PAH: Polycyclic aromatic hydrocarbons
MD: Molecular dynamics
QCM-D: Quartz crystal microbalance with dissipation monitoring
LUV: Large unilamellar vesicle
GUV: Giant unilamellar vesicles
LCSM: Laser confocal scanning microscope
DOPC: 1,2-Dioleoyl-sn-glycero-3-phosphocholine
DOPG: 1,2-Dioleoyl-sn-glycero-3-[phosphor-rac-(1-glycerol)]
DPPC: Dipalmitoyl phosphatidylcholine
POPG: Palmitoyloleoyl phosphatidylglycerol
HPLC: High performance liquid chromatography
CG: Coarse-grained
LJ: Lennard–Jones
SEM: Scanning electron microscope
TEM: Transmission electron microscope
AFM: Atomic force microscope
