# Supplementary material for: Competitive and/or cooperative interactions of graphene-family materials and benzo[a]pyrene with pulmonary surfactant: a computational and experimental study
Source: Part Fibre Toxicol. 2021 Dec 16;18:46. doi: 10.1186/s12989-021-00436-9 (PMC8675531; doi:10.1186/s12989-021-00436-9)
Supplement: Supplementary file 1 — Additional file 1: Text S1. Synthesis of graphene oxide. Text S2. SEM, TEM and AFM imaging of GFMs. Figure S1. Coarse-grained models of different component molecules and the simulation system setup. Figure S2. Simulated molecular agglomeration of BaPs in atmosphere. Figure S3. Effect of relative humidity on BaP agglomeration. Figure S4. Simulated adsorption of dispersed BaPs on graphene. Figure S5. Time sequences of typical snapshots depicting adsorption of agglomerated BaPs on graphene and the induced graphene curling. Figure S6. Effect of relative humidity on BaP adsorption on graphene. Figure S7. Deposition of a bare graphene nanosheet on the PS layer. Figure S8. Time evolutions of the energy of interactions between different components under different adsorption states. Figure S9. Solubilization of agglomerated BaPs by PS. Figure S10. The calculated mean square displacement (MSD) for different components of PS and the deposited BaPs of different numbers. Figure S11. Distinct orientations of BaPs respectively at the upper and lower surfaces of graphene deposited at the PS layer. Figure S12. Ultrastructure perturbation of PS induced by graphene adsorbed with 200 BaPs. Figure S13. Joint interactions between the PS layer and curled graphene with encapsulated BaPs. Figure S14. Final simulated snapshots from both top and bottom views and the local PS order parameter diagrams. Figure S15. Deposition of a bare graphene oxide nanosheet on the PS layer. Figure S16. Effects of graphene oxidation and BaP adsorption on joint interactions between graphene, BaP and PS. Figure S17. Detection of malondialdehyde. Figure S18. MD simulations of cell membrane interactions with GFMs adsorbed with BaPs. Figure S19. Typical snapshot showing distribution of PS molecules at the pore edge. Figure S20. PS extraction and layer damage induced by graphene as affected by graphene oxidation and BaP adsorption. Figure S21. The formation of LUV layer on the QCM-D sensor (Au sensor). Figure S22. S [file 12989_2021_436_MOESM1_ESM.doc]

**Supporting Information**

**Competitive and/or cooperative interactions of graphene-family materials and benzo[a]pyrene with pulmonary surfactant: A computational and experimental study**

Tongtao Yue^1,2^, Rujie Lv^3^, Dongfang Xu^1^, Yan Xu^4^, Lu Liu^1^, Yanhui Dai^1^, Jian Zhao^1,2*^ and Baoshan Xing^5*^

^1^ Institute of Coastal Environmental Pollution Control, Ministry of Education Key Laboratory of Marine Environment and Ecology, Frontiers Science Center for Deep Ocean Multispheres and Earth System, Ocean University of China, Qingdao 266100, China

^2^ Laboratory for Marine Ecology and Environmental Science, Qingdao National Laboratory for Marine Science and Technology, Qingdao 266237, China

^3^ College of Chemical Engineering, China University of Petroleum (East China), Qingdao 266580, China

^4^ College of Electronic Engineering and Automation, Shandong University of Science and Technology, Qingdao 266590, China

^5^ Stockbridge School of Agriculture, University of Massachusetts, Amherst, Massachusetts 01003, United States

^*^ Correspondence: [jzhao@ouc.edu.cn](mailto:jzhao@ouc.edu.cn); [bx@umass.edu](mailto:bx@umass.edu)

**Text S1:** **Synthesis of graphene oxide**

Graphene oxide used in our experiments was prepared using an improved synthesis method. Briefly, 0.75 g of graphite was added into a mixture of concentrated H_2_SO_4_/H_3_PO_4_ (90:10 mL, Fisher Scientific, USA) in an ice-bath. KMnO_4_ (4.5 g, Fisher Scientific, USA) was added with agitation, then transferred to 50 °C air bath and stirred for 24 h. 100 mL water-ice mixtures with 2 mL H_2_O_2_ (30%, Fisher Scientific, USA) were poured into systems. The mixture was cooled and centrifuged at 13500 rpm for 30 min (Sorvall super T21 benchtop centrifuge, Thermo Fisher Scientific, USA) to collect the sediment. The sediment was washed with HCl (10%, Fisher Scientific, USA) and DI water, then collected solid was freeze-dried for 48 h under vacuum. After that, it was dispersed in DI water by ultrasonication (Misonix sonicator, S-4000, 600 W, 50% amplitude) at a concentration of 250 mg/L to get a suspension. 200 mL suspension was mixed with 54 µl hydrazine (64-65 wt% in water, Sigma-Aldrich, USA) and 0.7 mL ammonia solution (28-30 wt% in water, Fisher Scientific, USA). After 1-h dry at 95 ℃, black suspension was filtered and washed with deionized water until the filtrate pH approached neutral. The resulting wet samples were kept in a 60 ℃ oven to remove bulk water and then transferred to a 220 ℃ muffle furnace to further remove the residual oxygen, and finally graphene oxide was synthesized.

**Text S2.** SEM, TEM and AFM imaging of GFMs.

The morphologies of GFMs were observed using scanning electron microscope (SEM, HITACHI S-4800, Japan). After sonication, the GFMs suspensions were dropped into a copper mesh and observed after dried using scanning transmission electron microscope (TEM, Gemini300, Zeiss, German). Furthermore, the layer structure and thickness of GFMs were measured by an atomic force microscope (AFM, Agilent 5400 AFM, Agilent, USA).


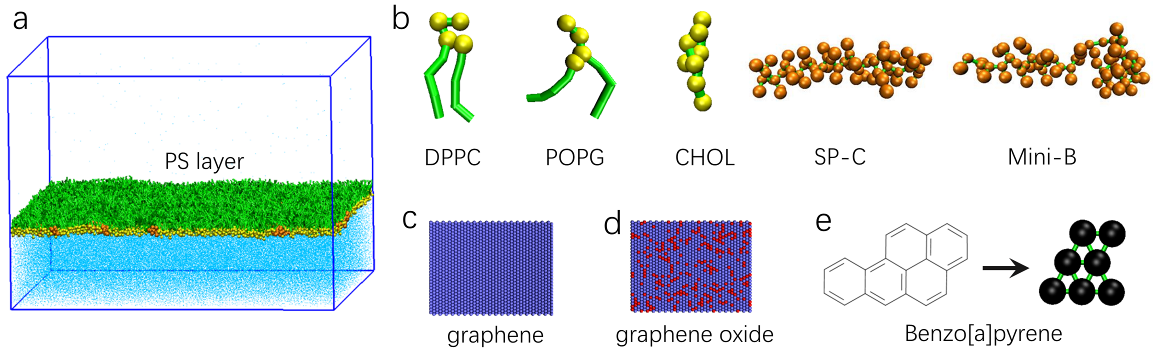


**Figure S1.** Coarse-grained models of different component molecules and the simulation system setup. (a) Multi-component PS lining at the air-water interface, with water molecules underneath the PS layer displayed in blue. (b) CG models of different PS components, including DPPC, POPG, cholesterol (CHOL), SP-C and Mini-B. (c, d) CG models of graphene (c) and graphene oxide (d) nanosheets with a lateral size of 10.8 nm × 14.4 nm. (e) All-atom and corresponding CG models of BaP used in our simulations.


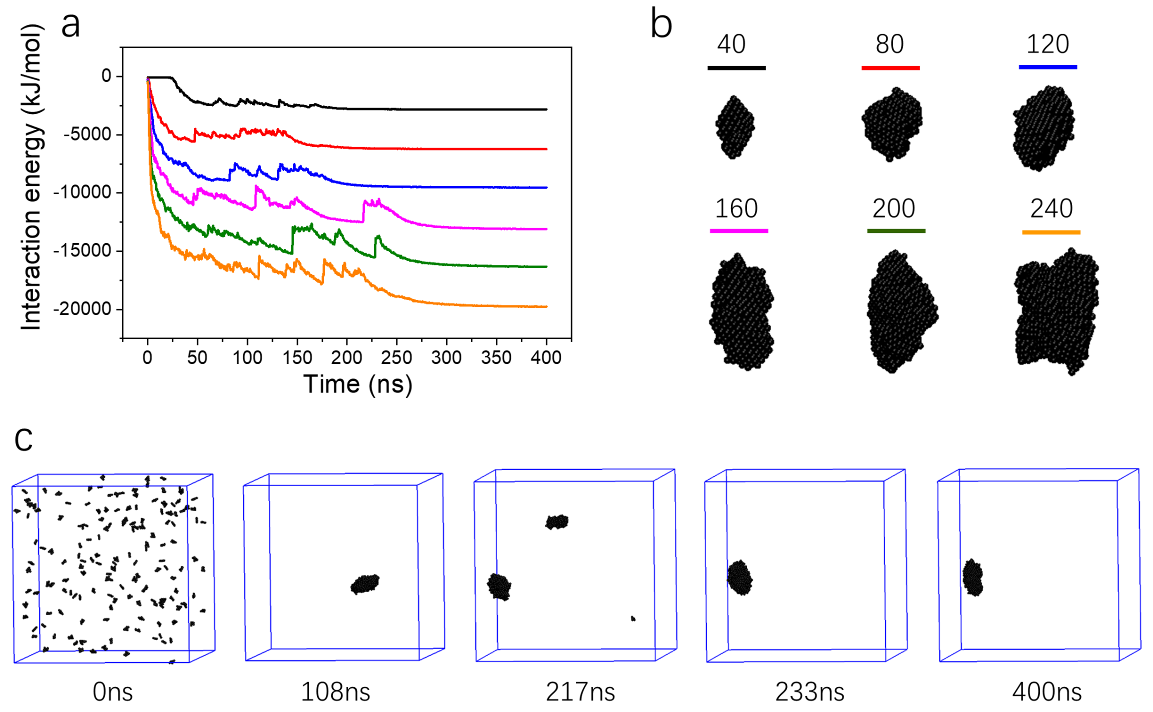


**Figure S2.** Simulated molecular agglomeration of BaPs in atmosphere. (a) Time evolutions of the BaP-BaP interaction energy during the gaseous agglomeration of BaPs of different numbers. (b) Final simulated molecular agglomerates of BaPs of different numbers. (c) Time sequence of typical snapshots depicting the process of molecular agglomeration of 160 BaPs in a gaseous box.


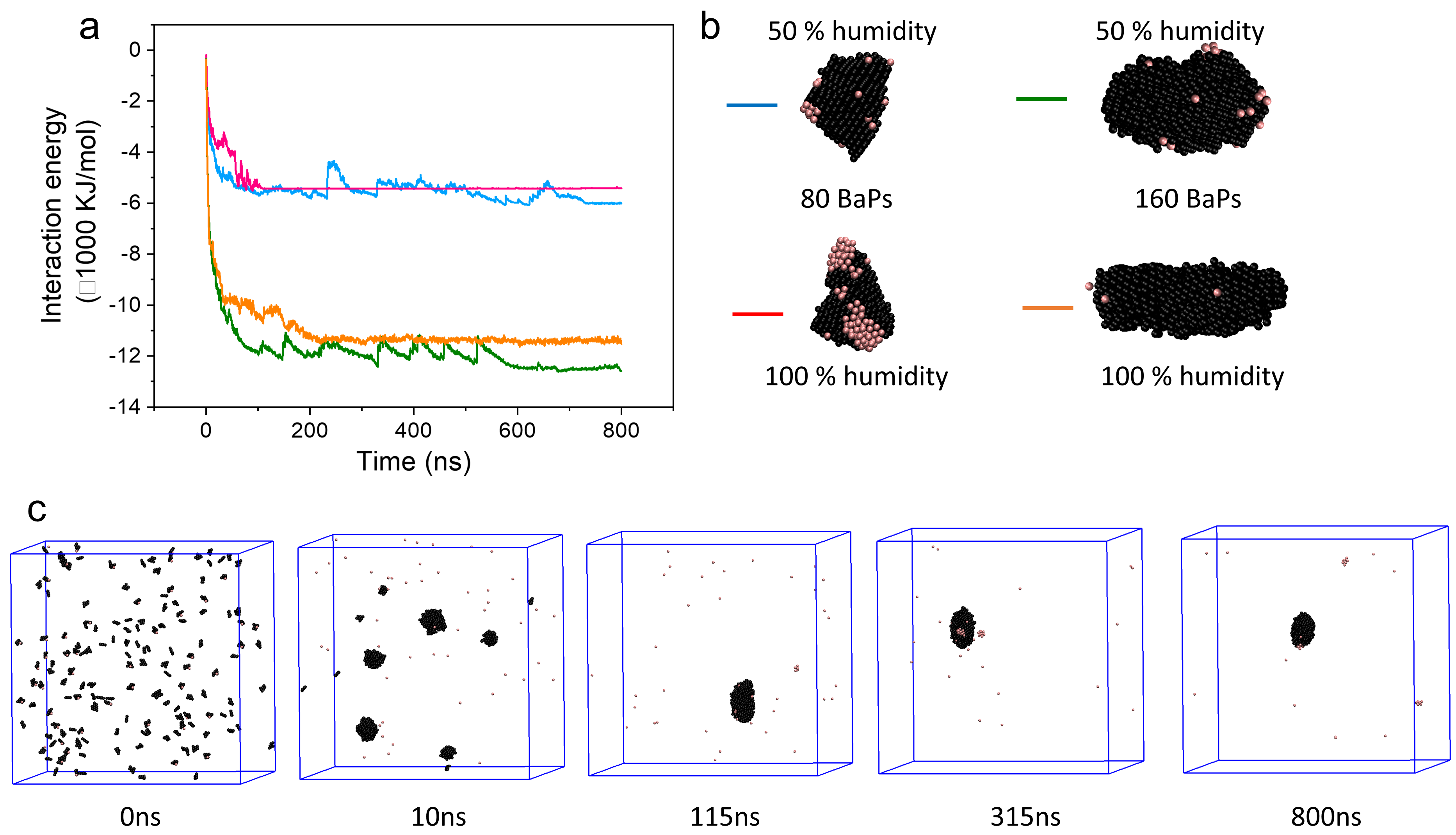


**Figure S3.** Effect of relative humidity on BaP agglomeration. (a) Time evolutions of the BaP-BaP interaction energy during the gaseous agglomeration of 80 and 160 BaPs at different relative humidity (50% and 100%). (b) Final simulated molecular agglomerates of 80 and 160 BaPs at relative humidity of 50 % and 100 %. Water beads are displayed in pink. (c) Time sequence of typical snapshots depicting agglomeration of 80 BaPs at the relative humidity of 50%.


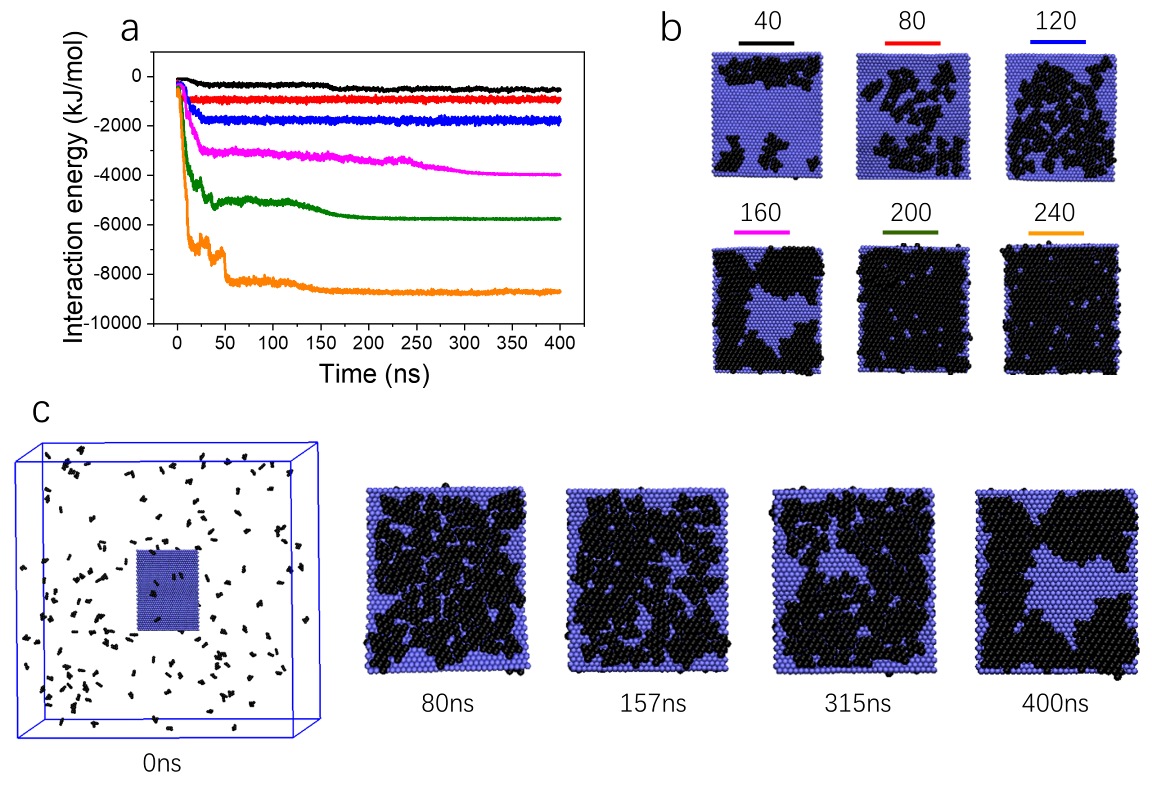


**Figure S4.** Simulated adsorption of dispersed BaPs on graphene. (a) Time evolutions of the BaP-graphene interaction energy under different BaP concentrations. (b) Final simulated snapshots of BaP adsorption on graphene under different BaP concentrations. (c) Time sequence of typical snapshots depicting adsorption of 160 BaPs on graphene. Combining the curves of the interaction energy, the whole process is divided into the BaP adsorption stage and the BaP rearrangement stage.


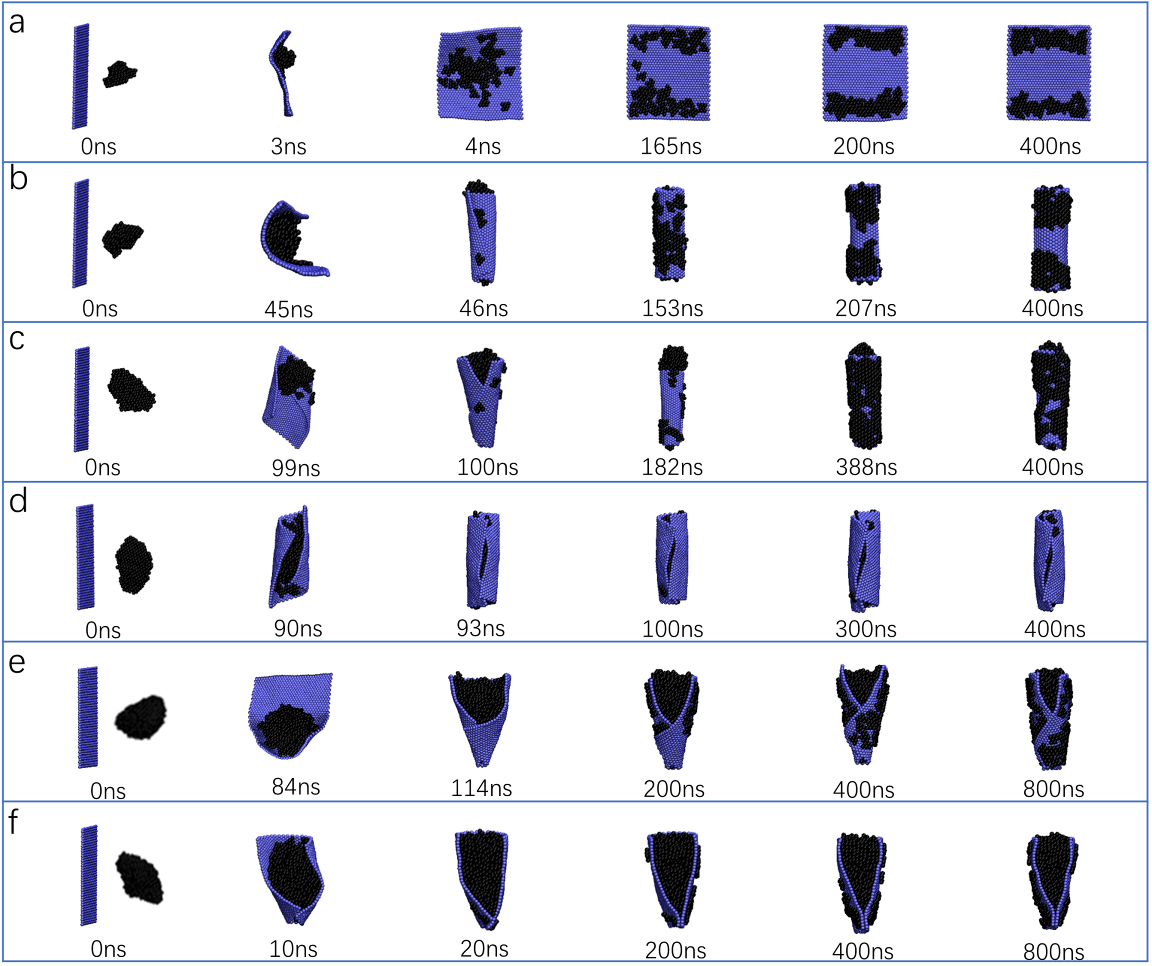


**Figure S5.** Time sequences of typical snapshots depicting adsorption of agglomerated BaPs on graphene and the induced graphene curling. The numbers of BaPs in one agglomerate are 40 (a), 80 (b), 120 (c), 160 (d), 200 (e) and 240 (f), respectively. As is seen, once the size of one BaP agglomerate exceeds a critical value that depends on the graphene nanosheet size, the adsorption of agglomerated BaPs causes the graphene nanosheet to curl and encapsulate BaPs.


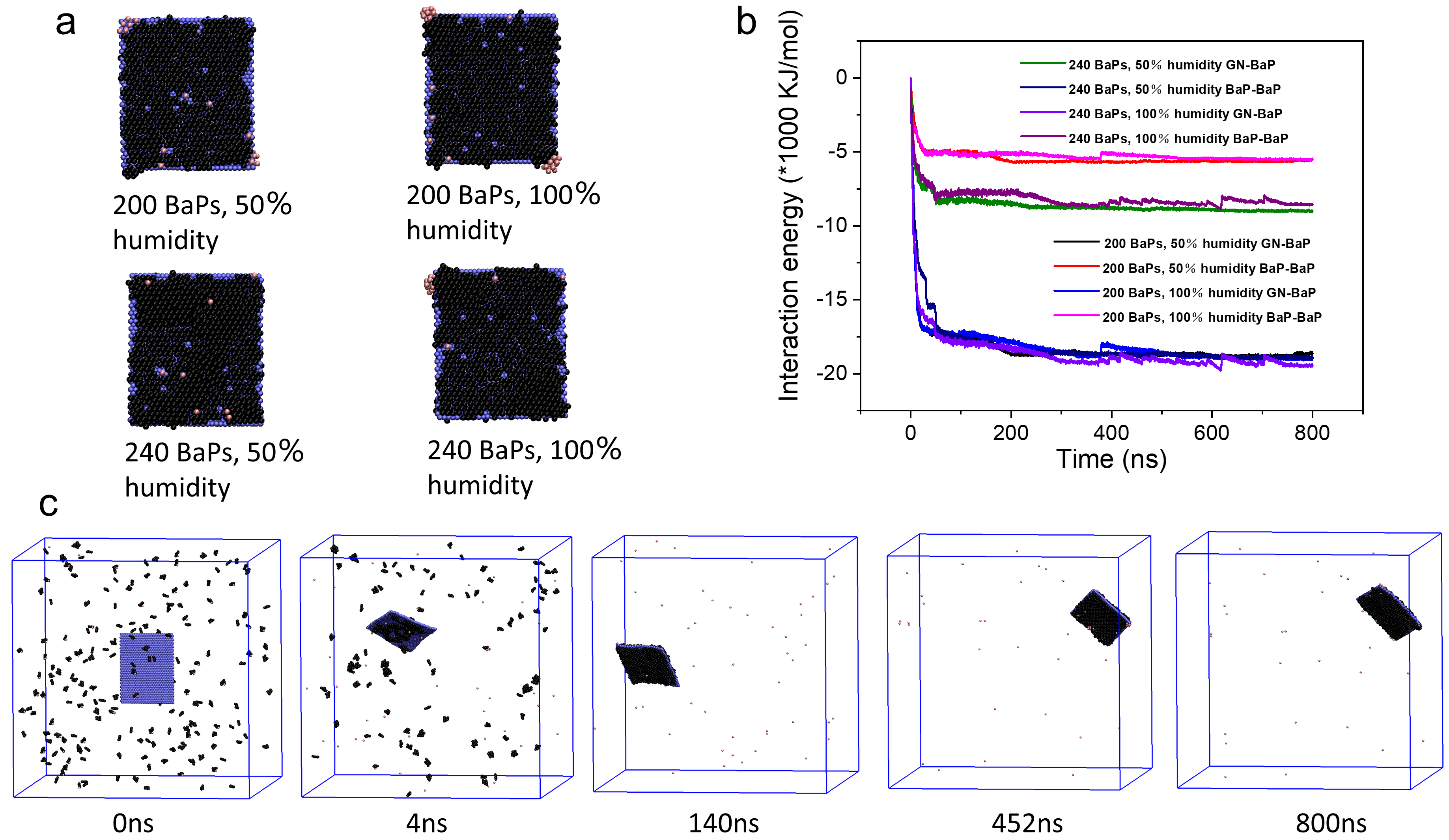


**Figure S6.** Effect of relative humidity on BaP adsorption on graphene. (a) Final simulated snapshots of 200 and 240 BaPs adsorbed on graphene at relative humidity of 50% and 100 %. (b) Time evolutions of the BaP-graphene interaction energy at different humidity. (c) Time sequence of snapshots depicting adsorption of 200 BaPs on graphene at a relative humidity of 50%.


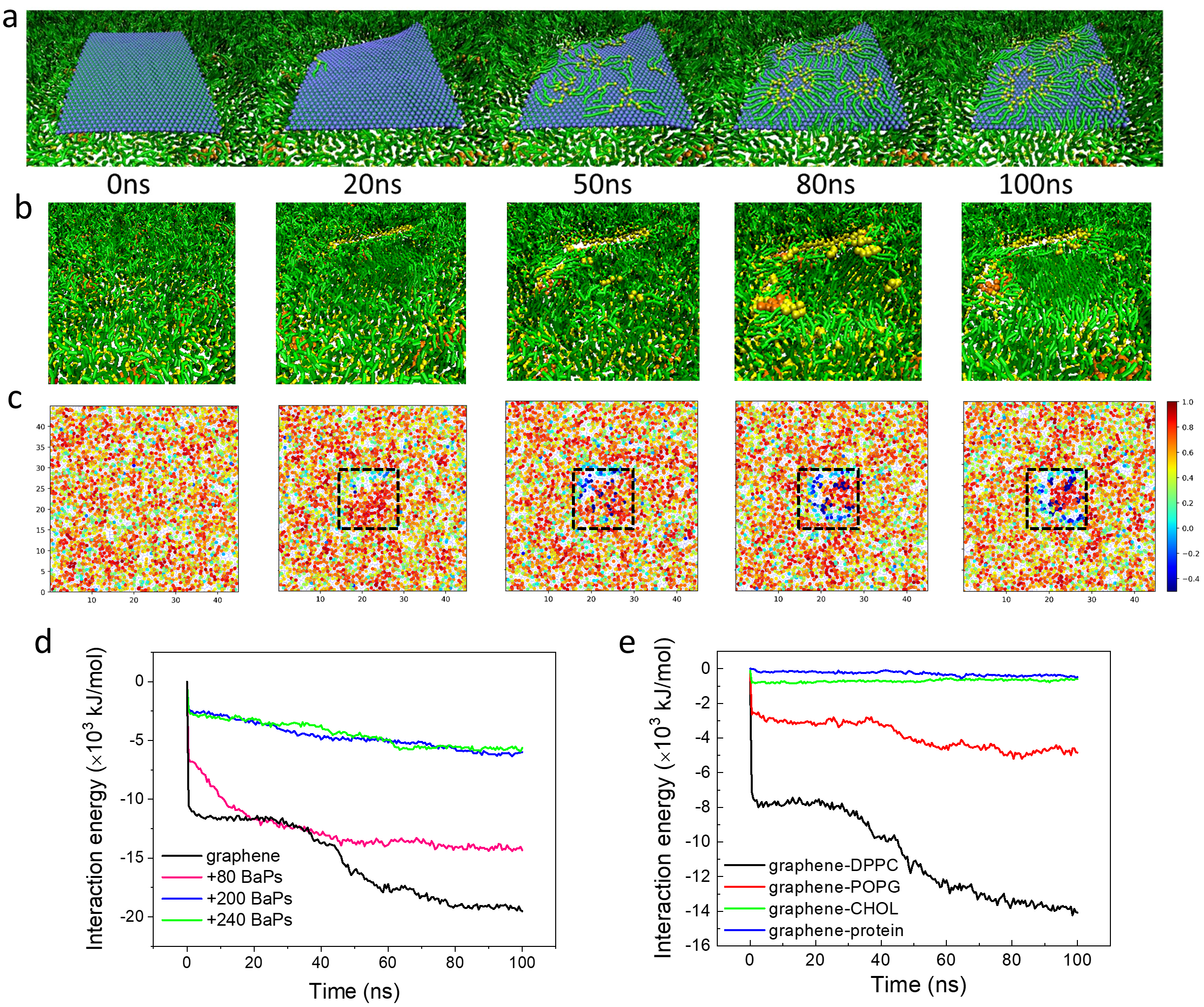


**Figure S7.** Deposition of a bare graphene nanosheet on the PS layer. (a) Time sequence of typical snapshots depicting adhesion of a bare graphene nanosheet on the PS layer followed by extraction of PS molecules to form inverse micelles on the upper graphene surface. (b) Local amplified structures with extracted PS molecules excluded for viewing PS perturbation underneath the graphene. (c) The calculated order parameters for lipids are used to color the Voronoi lattice, illustrating PS arrangement perturbed by graphene. The location of graphene is labeled with the black dashed square. (d) Time evolutions of the graphene-PS interaction energy under different numbers of BaP adsorbed. (e) Time evolutions of graphene interactions with different PS component molecules.


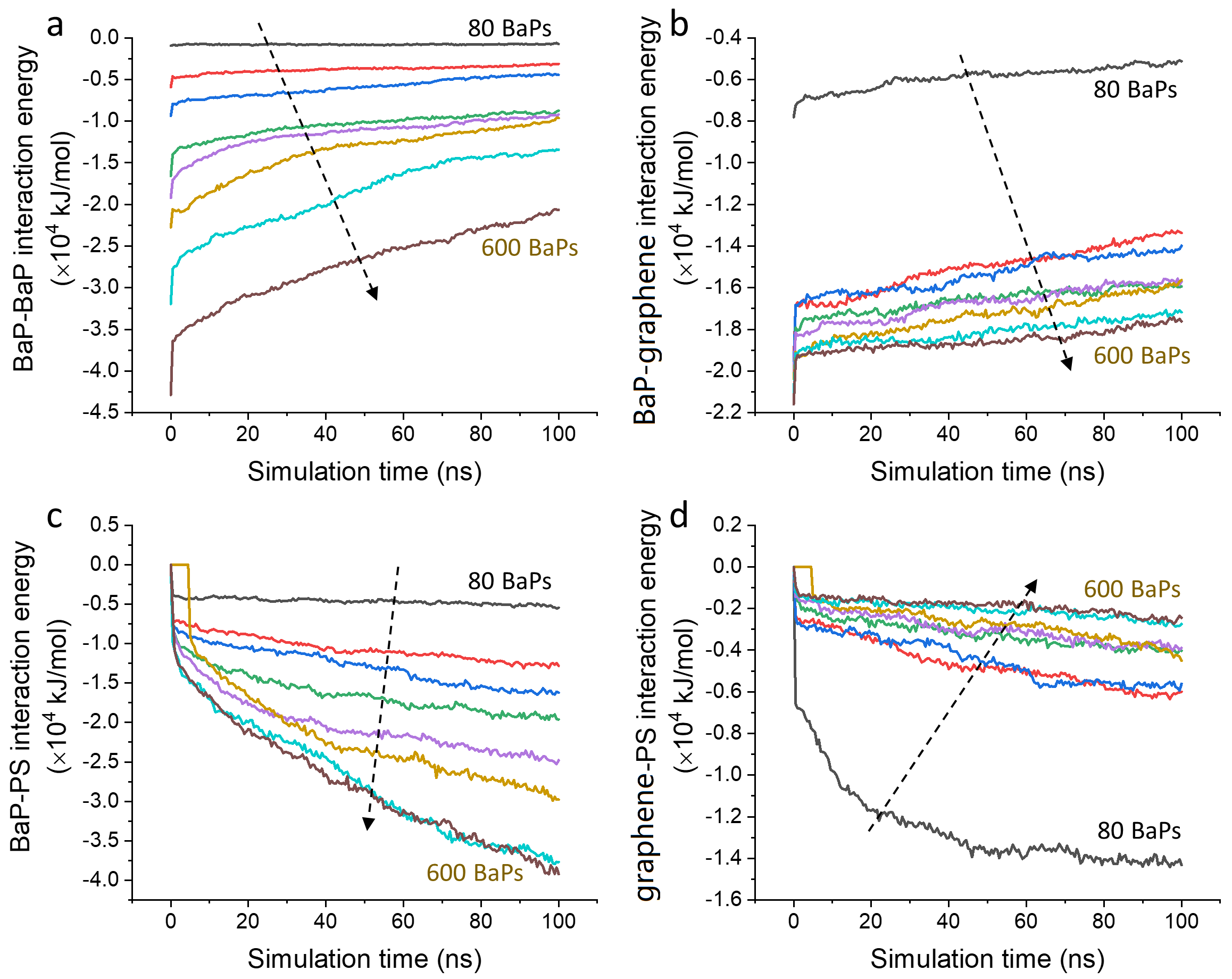
**Figure S8.** Time evolutions of the BaP-BaP (a), BaP-graphene (b), BaP-PS (c), and graphene-PS (d) interaction energies under different adsorption states, including partial adsorption state, saturate adsorption state and multi-layered adsorption state.


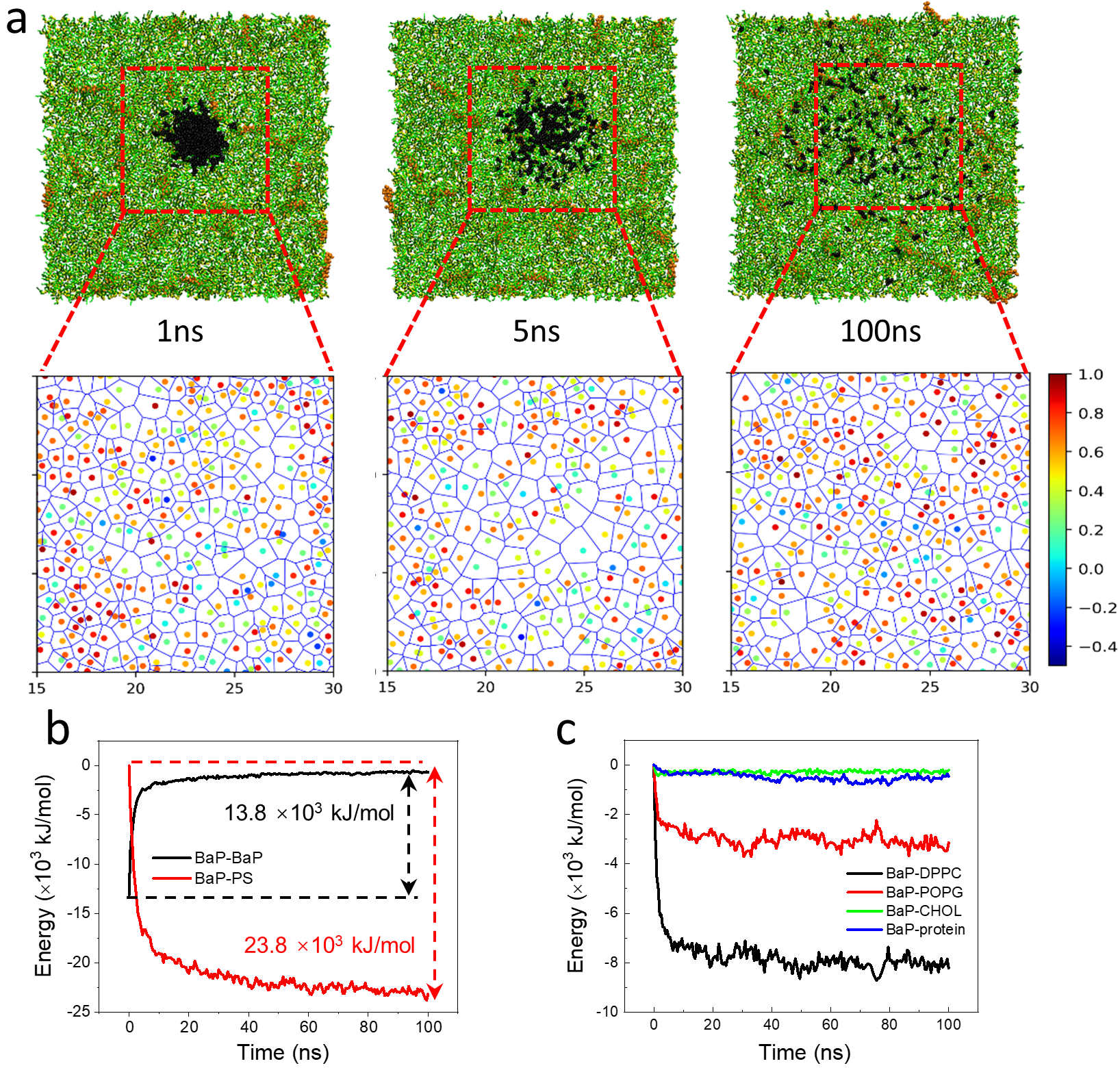


**Figure S9.** Solubilization of agglomerated BaPs by PS. (a) Time sequence of typical snapshots. The calculated order parameters for PS component of lipids are used to color the Voronoi lattice for view of PS perturbation by BaPs. The transient PS perturbation by deposition of BaPs was labelled with a black dashed line in the Voronoi figure at t = 5 ns. (b) Time evolutions of both the BaP-BaP and BaP-PS interaction energies. (c) Time evolutions of the energy of BaP interactions with different PS components.


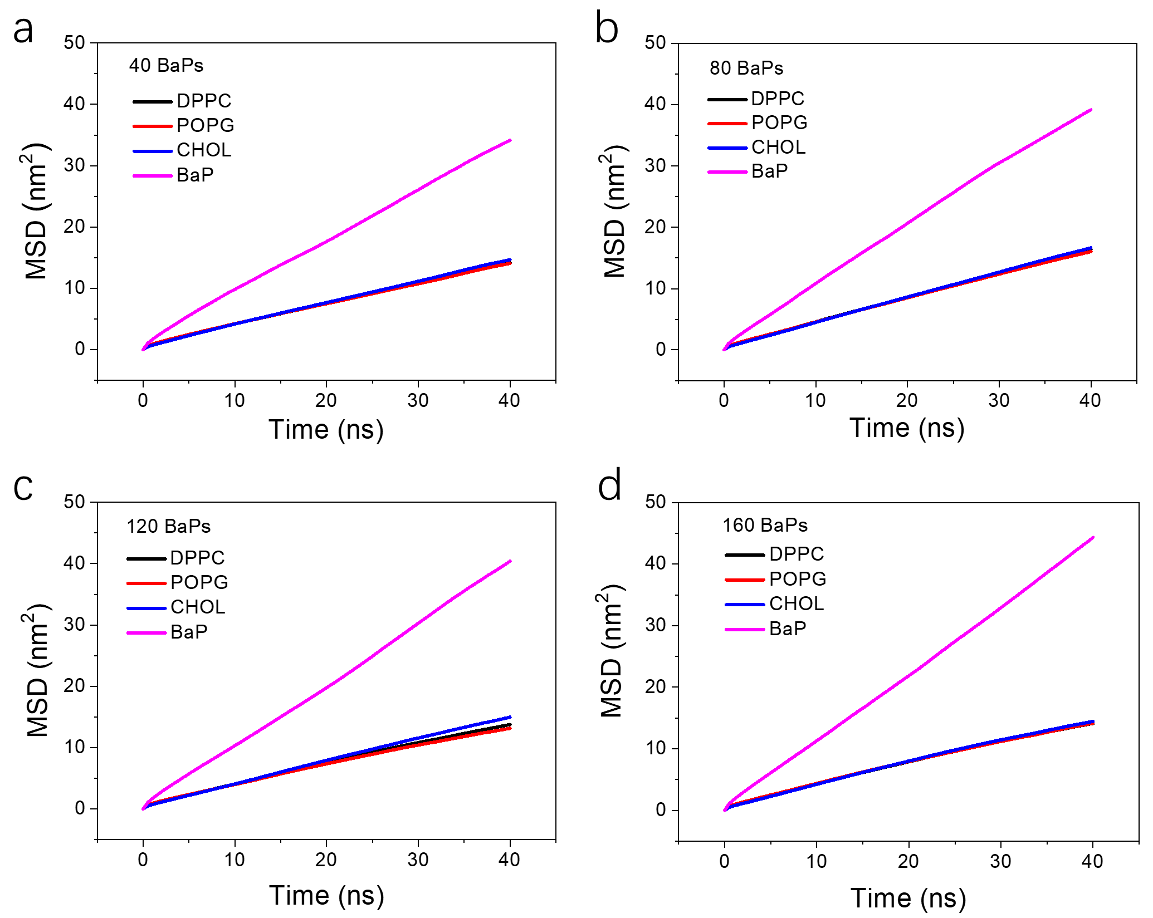


**Figure S10.** The calculated mean square displacement (MSD) for different components of PS and the deposited BaPs of different numbers. The numbers of BaPs are 40 (a), 80 (b), 120 (c) and 160 (d), respectively.


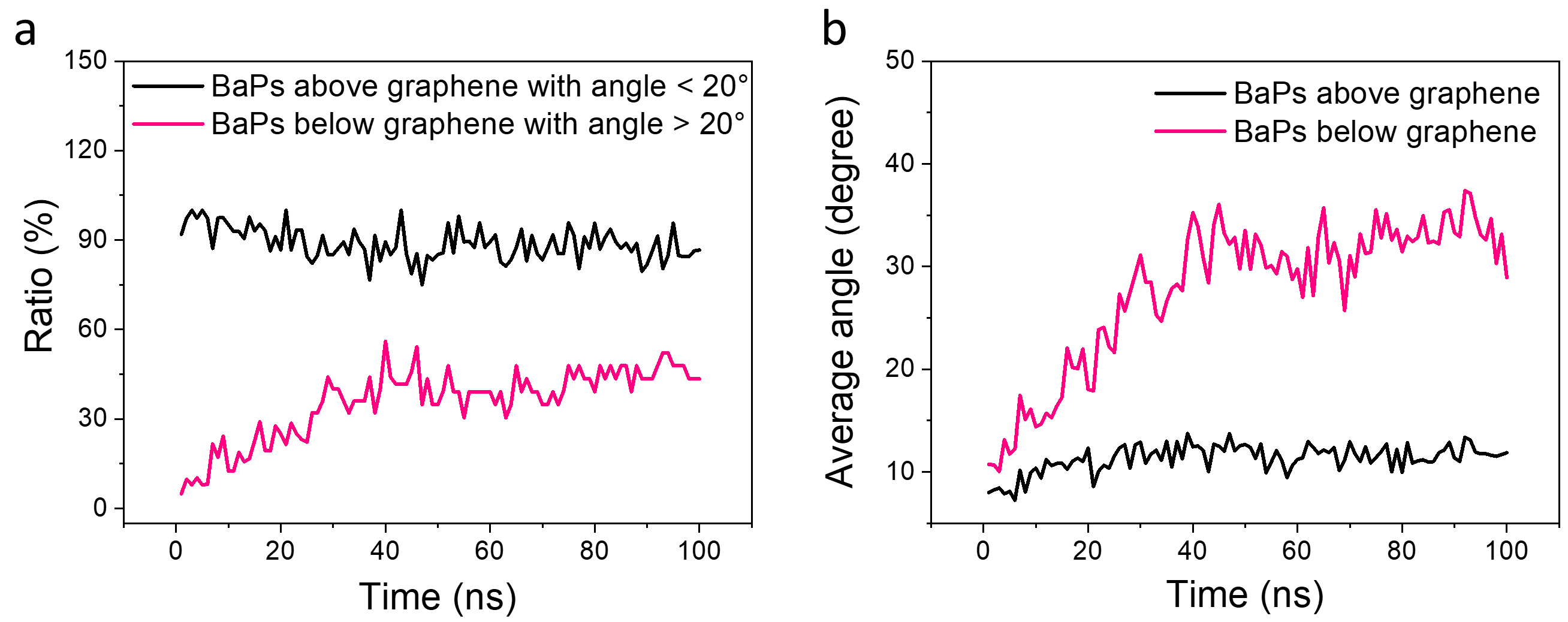


**Figure S11.** Distinct orientations of BaPs adsorbed respectively above and below graphene deposited at the PS layer. (a) Time evolutions of the ratio of BaPs exhibiting small (< 20°) and large (> 20°) tilt angles with respect to the PS layer plane. (b) Time evolutions of the average angle for BaPs at respectively above and below the graphene.


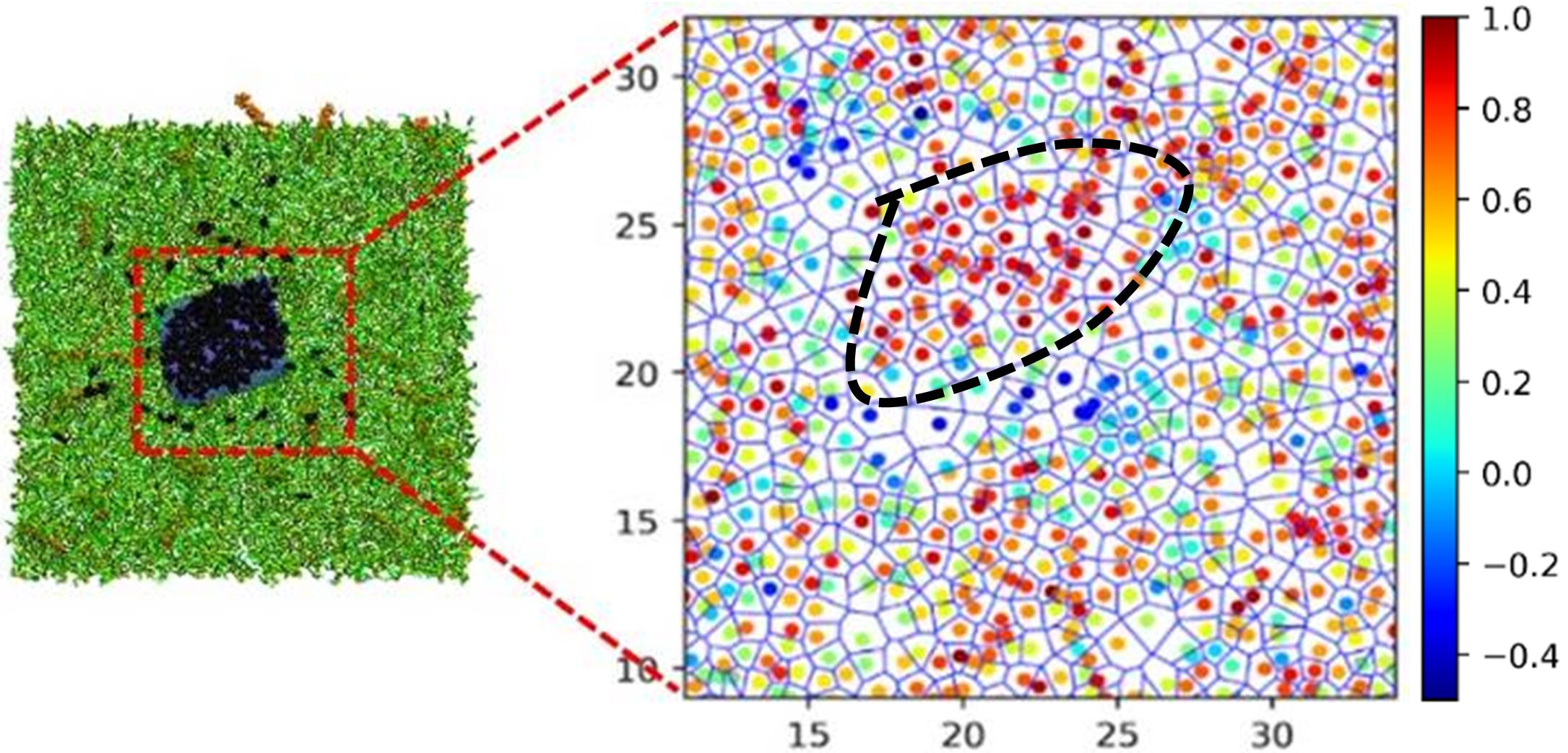


**Figure S12.** Ultrastructure perturbation of PS induced by graphene adsorbed with 200 BaPs. The left panel is the snapshot from the top view, and the right panel is the enlarged Voronoi lattice for viewing the order parameters of lipid molecules around graphene. The higher ordering of lipids beneath the graphene due to detachment of BaPs was labelled with a black dashed line in the Voronoi figure.


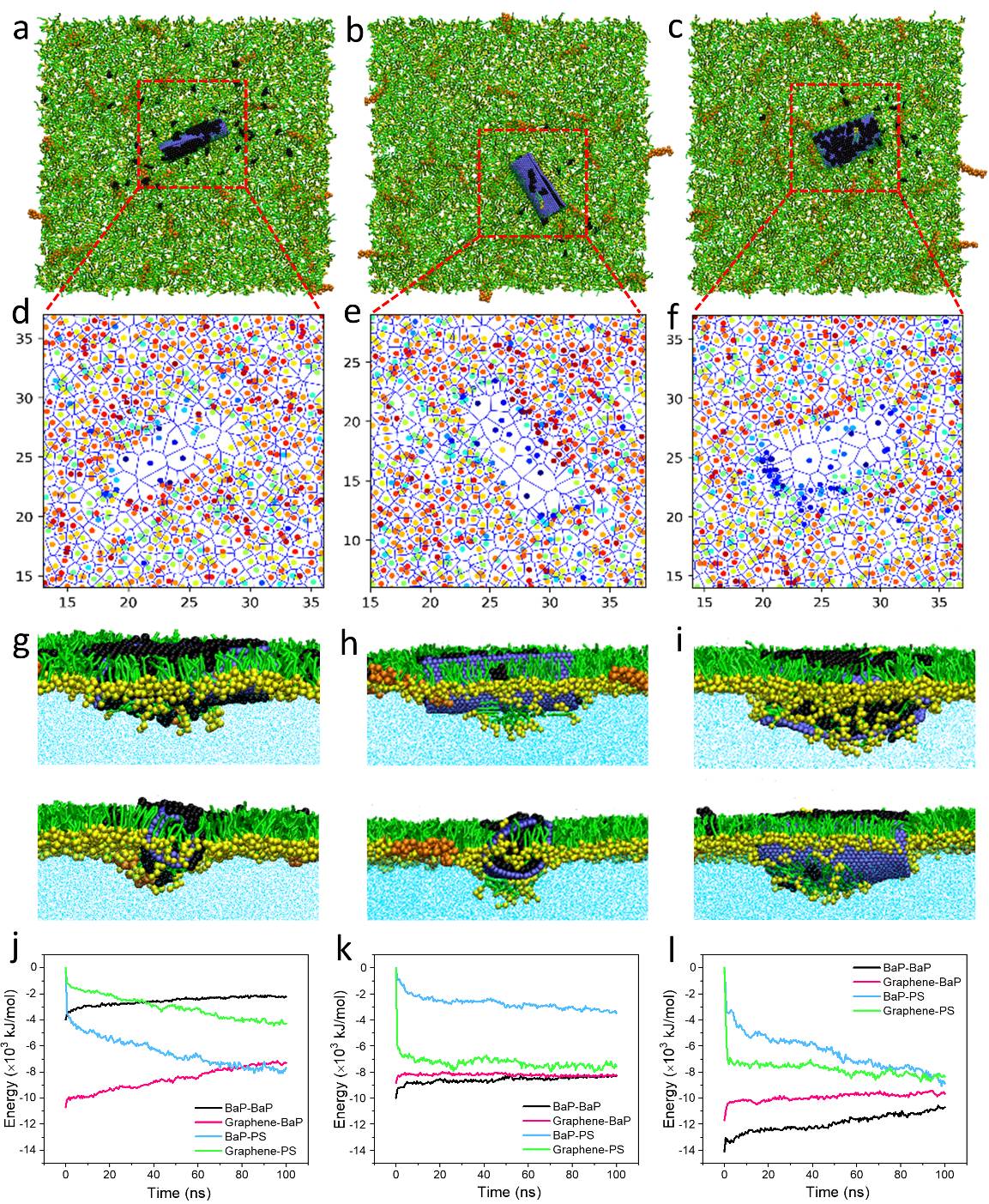


**Figure S13.** Joint interactions between the PS layer and curled graphene with encapsulated BaPs. (a-c) Final snapshots of PS interactions with the tubular graphene-BaP nanostructures from the top view. (d-f) Localized distribution of lipid order parameter around deposited graphene. (g-i) Locally enlarged structures from the cross-sectional view illustrating rearrangement of PS molecules around the complexes. (j-l) Time evolutions of the BaP-BaP, BaP-graphene, BaP-PS and graphene-PS interaction energies. The number of BaPs adsorbed on graphene is 120 (a, d, g, j), 160 (b, e, h, k) and 240 (c, f, i, l), respectively.


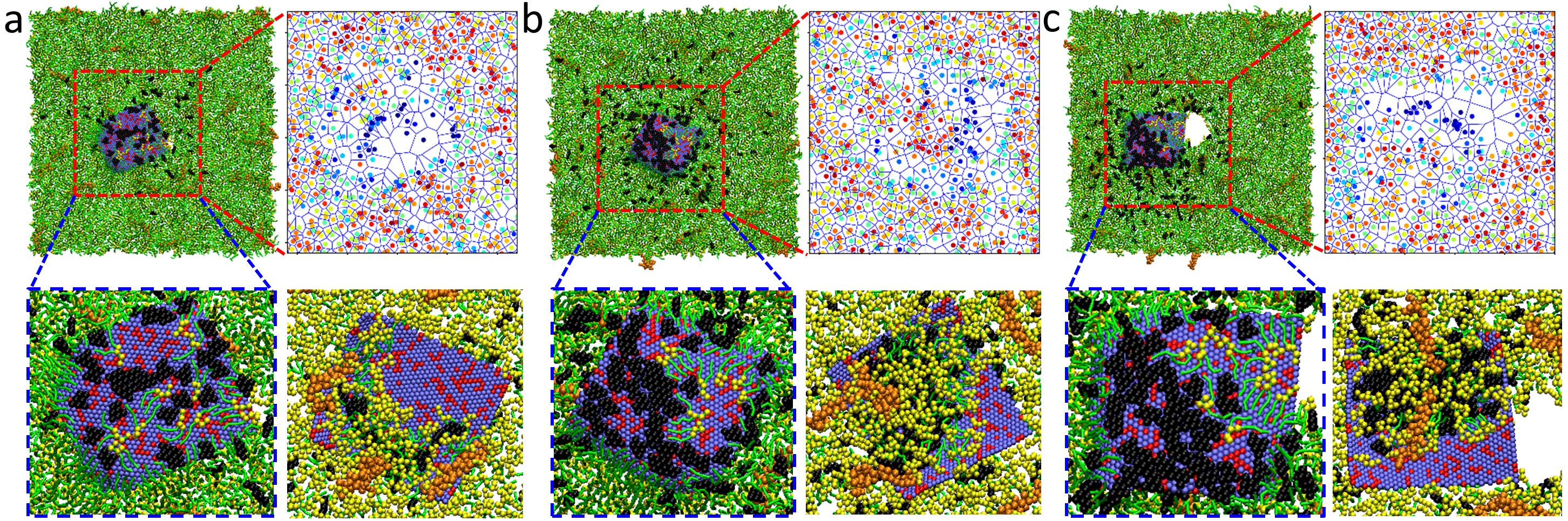


**Figure S14.** Final simulated snapshots from both top and bottom views and the local PS order parameter diagrams. The number of BaPs adsorbed on GO is respectively 80 (a), 200 (b) and 240 (c).

**
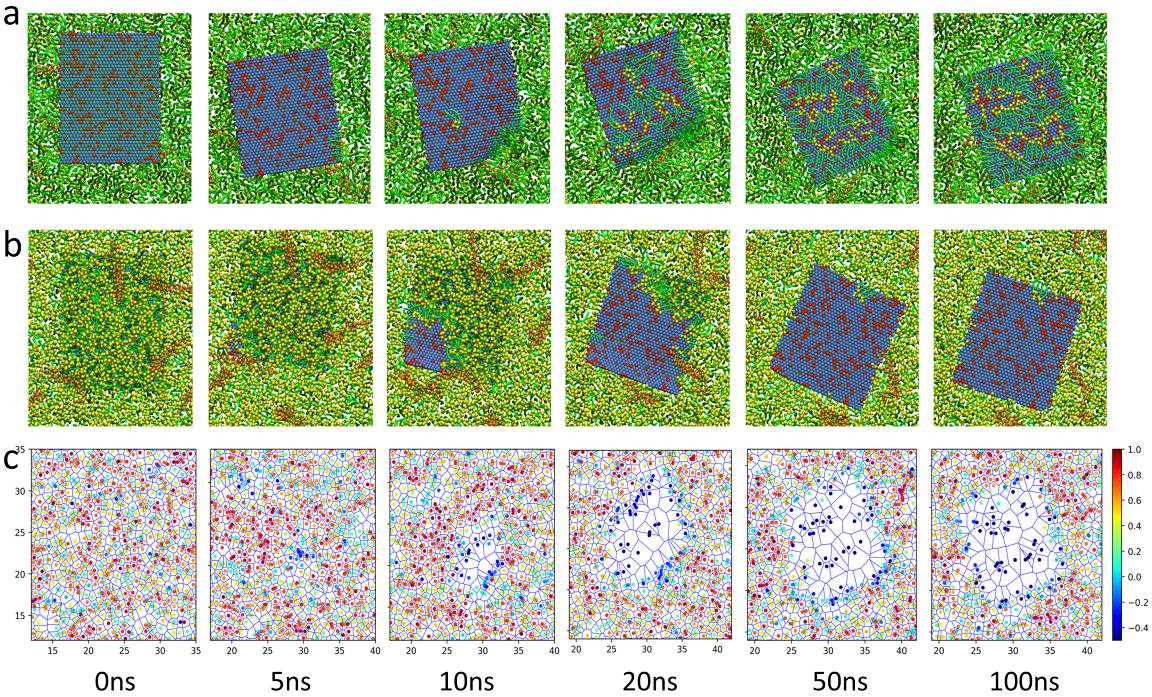
**

**Figure S15.** Deposition of a bare graphene oxide nanosheet on the PS layer. (a) Time sequence of the typical snapshot from the top view, illustrating the PS extraction and inverse micelle formation on the top surface of graphene oxide nanosheet. (b) Time sequence of the typical snapshot from the bottom view, illustrating rapid translocation of graphene oxide nanosheet across the PS layer. (c) Time sequence of the lipid order parameter diagram showing pore formation induced by the graphene oxide nanosheet.


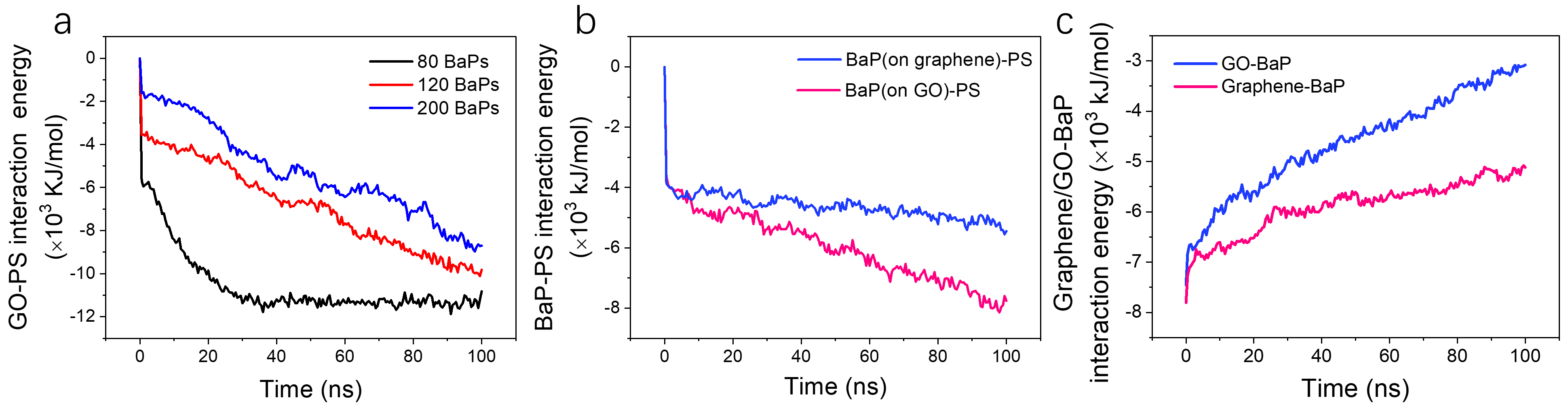


**Figure S16.** Effects of graphene oxidation and BaP adsorption on joint interactions between graphene, BaP and PS. (a) Time evolutions of the GO-PS interaction energy as influenced by adsorbed BaPs of different numbers. (b) Time evolutions of the interaction energy between PS and 80 BaPs adsorbed on graphene and graphene oxide. (c) Time evolutions of the interaction energy between graphene/GO and 80 BaPs.


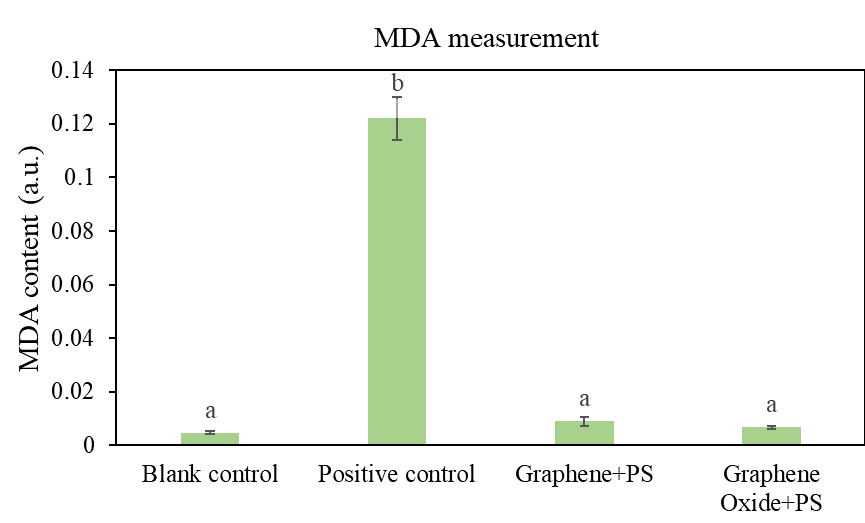


**Figure S17.** Malondialdehyde (MDA) content after incubation of PS with graphene and graphene oxide. The absorbance at 532 nm was measured to quantitively reflect the generation of MDA, which is typical production of PS oxidation. The concentrations of graphene/graphene oxide and PS were 50 mg/L and 40 mg/L, respectively.


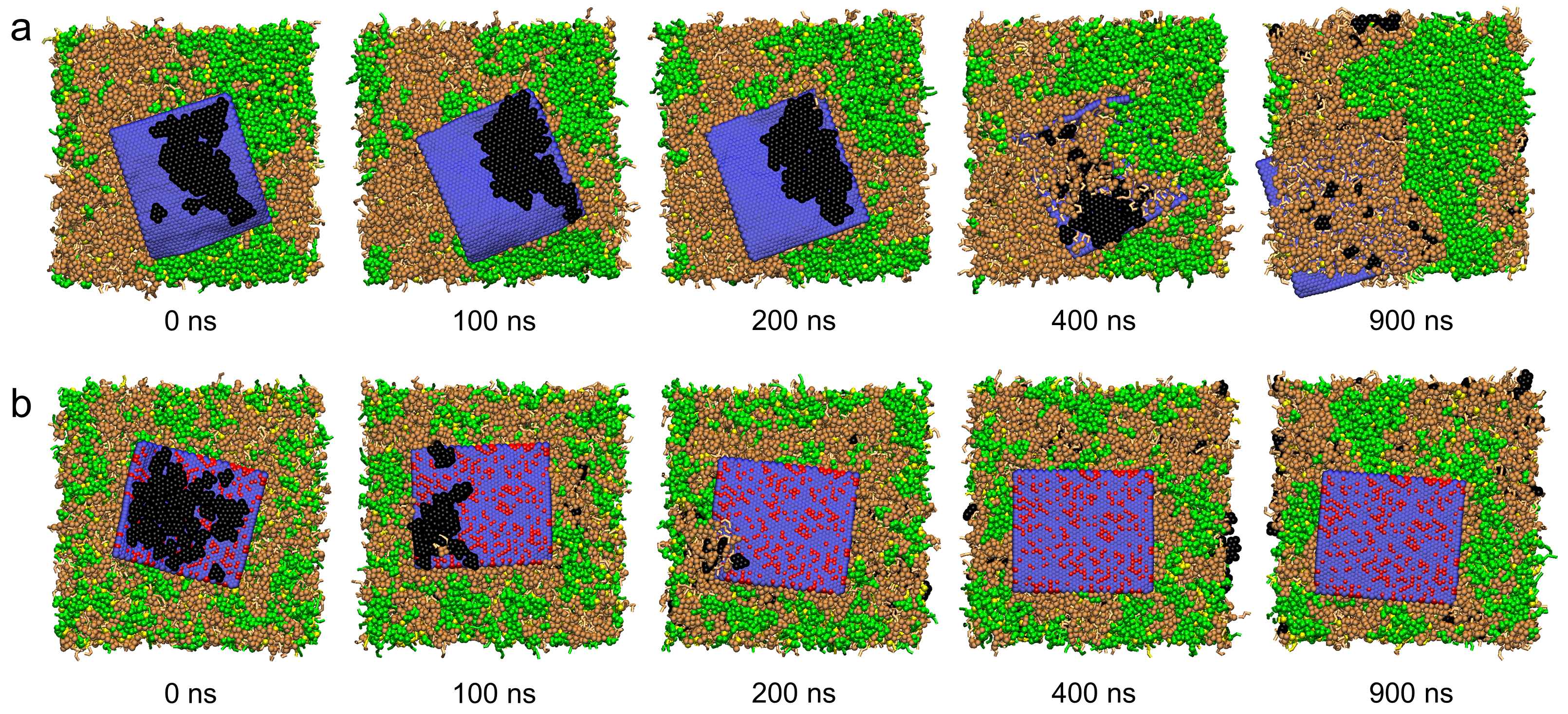


**Figure S18.** MD simulations of cell membrane interactions with GFMs adsorbed with BaPs. (a) Time sequence of typical snapshots of cell membrane interactions with graphene adsorbed with 80 BaPs. (b) Time sequence of typical snapshots of cell membrane interactions with graphene oxide adsorbed with 80 BaPs. Graphene is shown as blue nanosheet, with red dots representing oxidation. BaPs are colored in black. Saturated DPPC and unsaturated DIPC were colored in green and orange, respectively. Water molecules were not displayed for clarity.


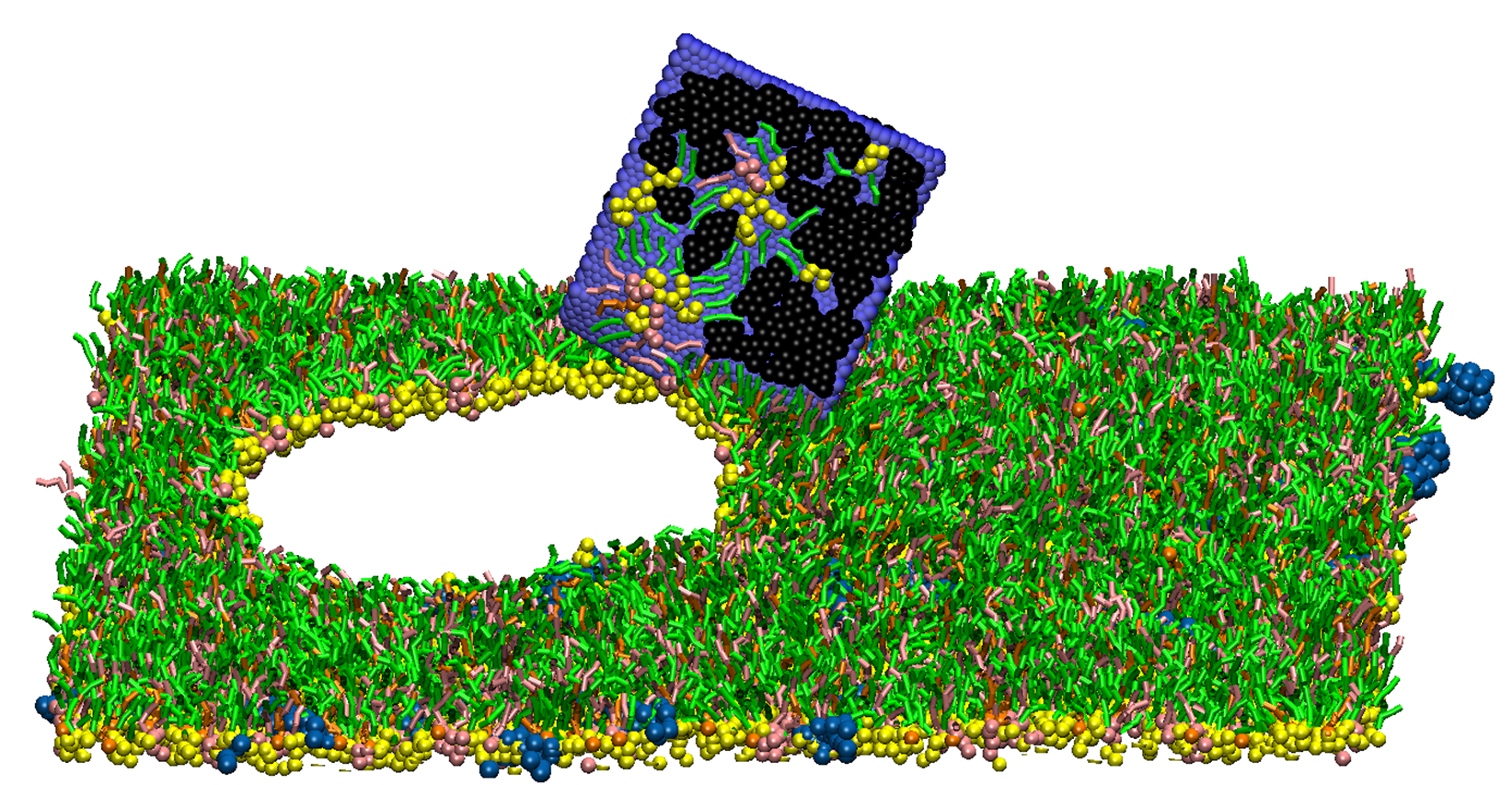


**Figure S19.** Side view of the PS extraction and layer rupture induced by suspended graphene nanosheet adsorbed with 80 BaPs. Headgroups of DPPC and POPG molecules were colored in yellow and pink for clarity of their distributions along the pore edge.


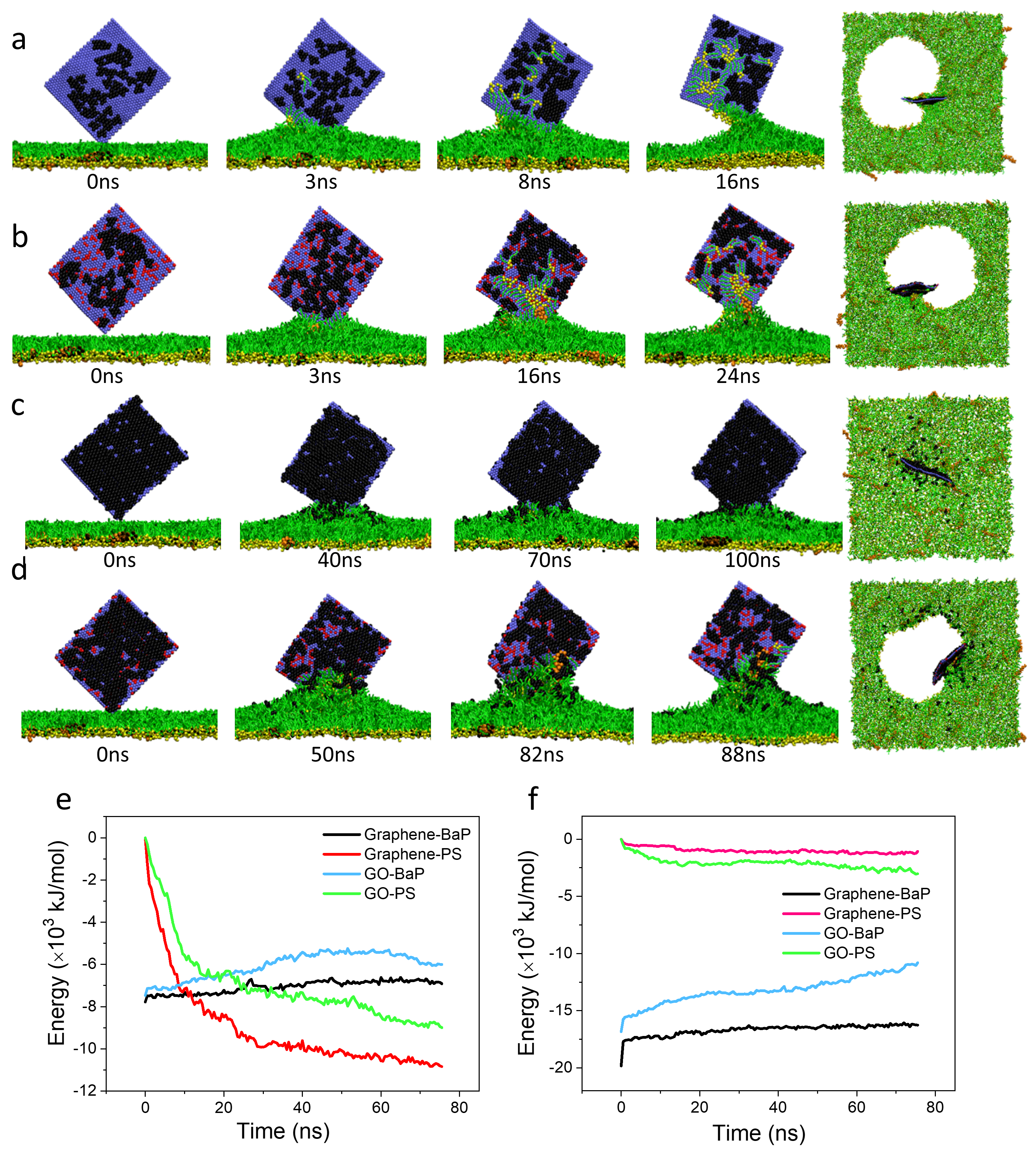


**Figure S20.** PS extraction and layer damage induced by graphene as affected by graphene oxidation and BaP adsorption. (a) Time sequence of typical snapshots of PS extraction by suspended graphene adsorbed with 80 BaPs. (b) PS extraction by graphene oxide adsorbed with 80 BaPs. (c) Graphene adsorbed with 200 BaPs shows no PS extraction. (d) GO adsorbed 200 BaPs can still extract PS via releasing BaPs into the layer. (e) Time evolutions of the interaction energies between different components as 80 (f) and 200 (g) BaPs were adsorbed on the suspended graphene and GO surfaces.


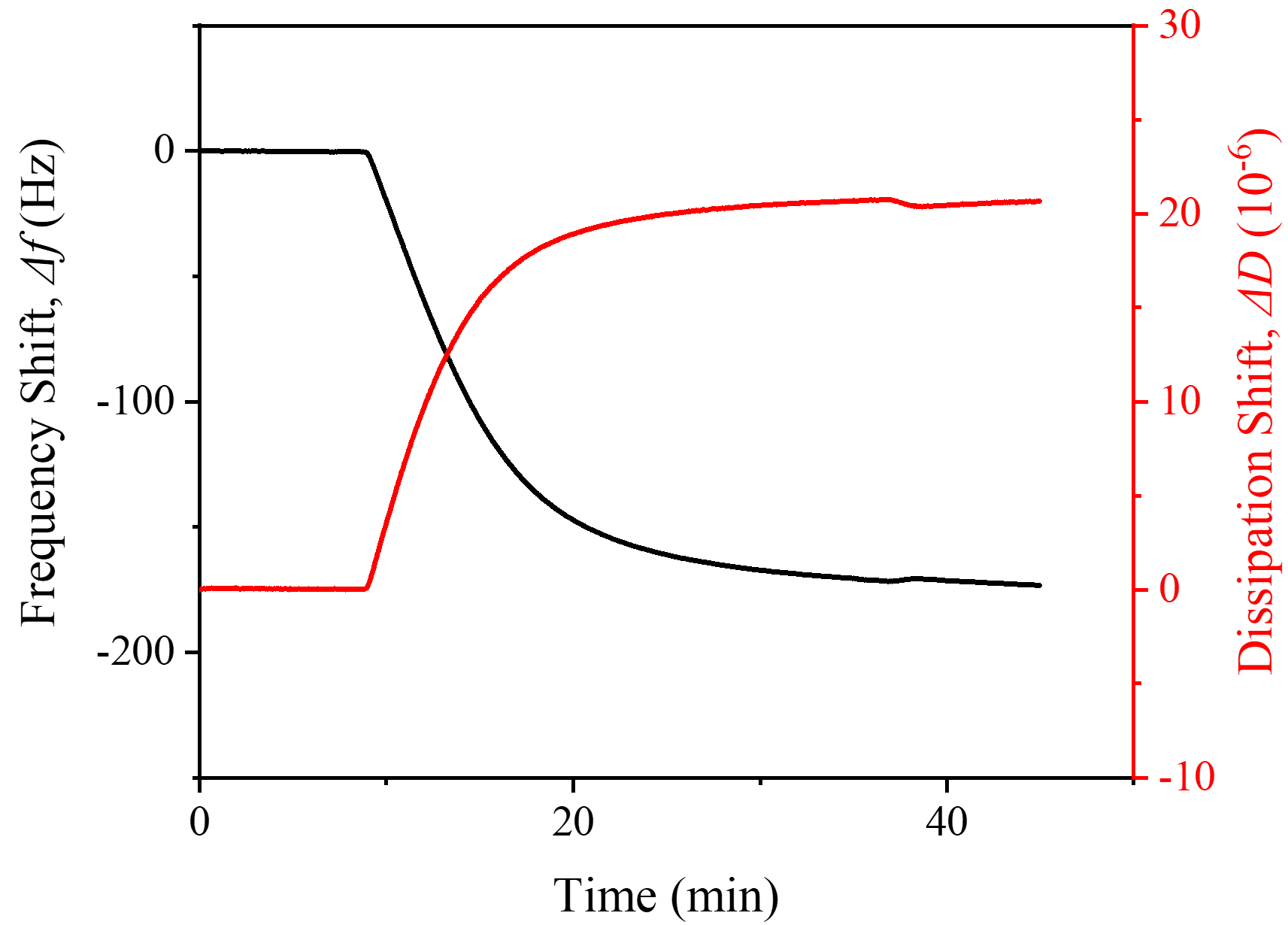


**Figure S21.** The formation of SUV layer on the QCM-D sensor (Au sensor). The changes of frequency shift (*∆F*) and dissipation shift (*∆D*) show the formation of SUV layer.


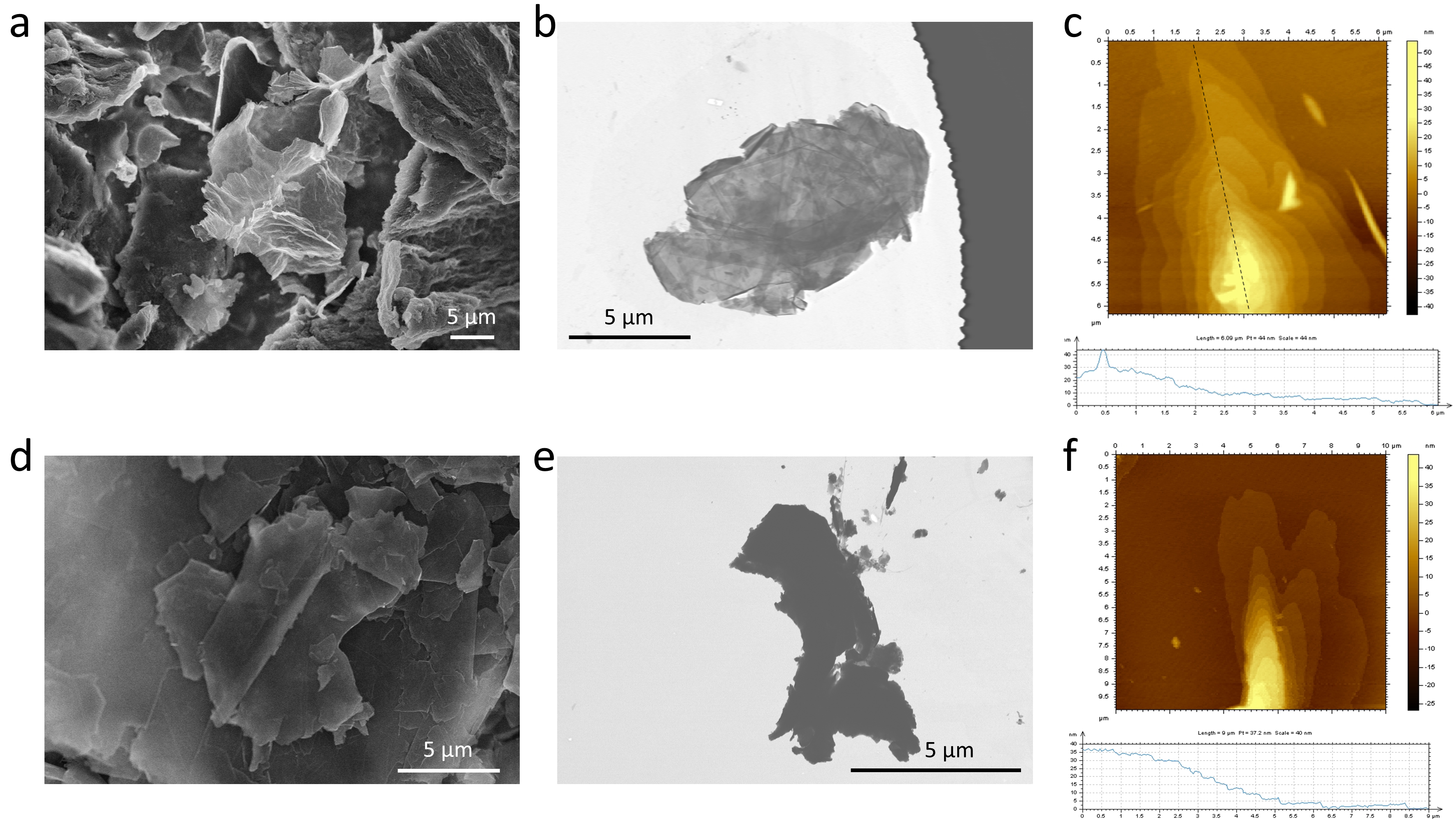


**Figure S22.** Scanning electron microscope (SEM) (a, d), transmission electron microscope (TEM) (b, e), and atomic force microscope (AFM) (c, f) imaging of graphene (a-c) and graphene oxide (d-f) used in experiments.


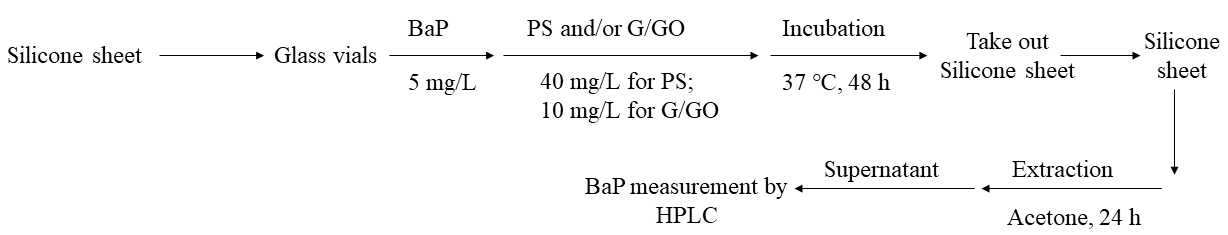


**Figure S23.** Schematic illustration of the solubilization experiment procedure.
